# Structures of SAS-6 coiled coil hold implications for the polarity of the centriolar cartwheel

**DOI:** 10.1016/j.str.2022.02.005

**Published:** 2022-05-05

**Authors:** Anastassia L. Kantsadi, Georgios N. Hatzopoulos, Pierre Gönczy, Ioannis Vakonakis

**Affiliations:** 1 Department of Biochemistry, University of Oxford, Oxford OX1 3QU, UK; 2 Swiss Institute for Experimental Cancer Research (ISREC), School of Life Sciences, Swiss Federal Institute of Technology Lausanne (EPFL), 1005 Lausanne, Switzerland

**Keywords:** centriole, cartwheel, SAS-6, coiled-coil, stacking, crystallography, electron microscopy, self-assembly, complex, asymmetry

## Abstract

Centrioles are eukaryotic organelles that template the formation of cilia and flagella, as well as organize the microtubule network and the mitotic spindle in animal cells. Centrioles have proximal-distal polarity and a 9-fold radial symmetry imparted by a likewise symmetrical central scaffold, the cartwheel. The spindle assembly abnormal protein 6 (SAS-6) self-assembles into 9-fold radially symmetric ring-shaped oligomers that stack via an unknown mechanism to form the cartwheel. Here, we uncover a homo-oligomerization interaction mediated by the coiled-coil domain of SAS-6. Crystallographic structures of *Chlamydomonas reinhardtii* SAS-6 coiled-coil complexes suggest this interaction is asymmetric, thereby imparting polarity to the cartwheel. Using a cryoelectron microscopy (cryo-EM) reconstitution assay, we demonstrate that amino acid substitutions disrupting this asymmetric association also impair SAS-6 ring stacking. Our work raises the possibility that the asymmetric interaction inherent to SAS-6 coiled-coil provides a polar element for cartwheel assembly, which may assist the establishment of the centriolar proximal-distal axis.

## Highlights

• CrSAS-6 coiled-coil domain forms higher-order oligomers in solution and crystals
• CrSAS-6 coiled-coil complexes exhibit symmetric and asymmetric configurations
• Asymmetric CrSAS-6 coiled-coil complex is important for stacking to form cartwheel
• Asymmetric CrSAS-6 coiled-coil could provide polarity to cartwheel assembly

## Graphical abstract

**Figure:**
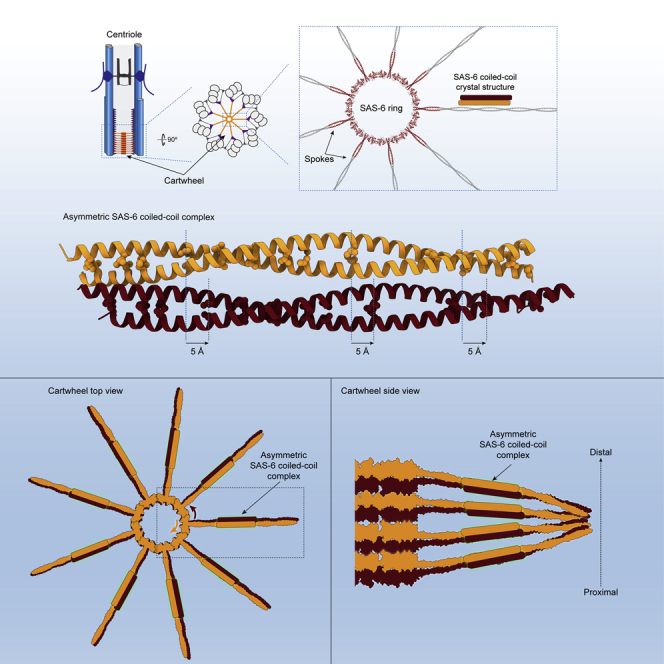

## Teaser

The centriolar protein SAS-6 self-assembles into 9-fold radially symmetric rings that stack to form the cartwheel, which might impart symmetry and chirality to the centriole. Kantsadi et al. uncover an asymmetric homo-oligomerization interaction mediated by the coiled-coil domain of SAS-6 that may help establishing the proximal-distal polarity of the centriole

## Introduction

Centrioles are cylindrical organelles found across the eukaryotic kingdom (Carvalho-Santos et al., 2011; Hodges et al., 2010; Marshall, 2009; Winey and O'Toole, 2014). In most proliferating animal cells, early in the cell cycle, a pair of centriolar cylinders surrounded by electron-dense pericentriolar material comprises the centrosome, which is located close to the nucleus and provides the major microtubule organizing center of the cell (Bornens, 2012; Conduit et al., 2015). During mitosis, the two centrosomes present at that stage of the cell cycle, each comprising two centriolar units, direct the formation of a bipolar spindle, thus contributing to the correct segregation of chromosomes. In animal cells that have exited the cell cycle, and broadly in unicellular eukaryotes, centrioles dock to the plasma membrane where they serve as basal bodies that template motile cilia and flagella, as well as the sensory primary cilium (Marshall, 2008). In this role, centrioles are essential for swimming cellular motility, such as in sperm, cellular signaling, and sensing of the surroundings.

Formation of new centrioles in the canonical duplication cycle occurs at a single site adjacent to each pre-existing centriole, starting in late G1/early S phases of the cell cycle (reviewed in Azimzadeh and Marshall, 2010; Firat-Karalar and Stearns, 2014; Gönczy and Hatzopoulos, 2019; Jana et al., 2014). The first structural component localizing at the centriole assembly site is the spindle assembly abnormal protein 6 (SAS-6). SAS-6 localization relies on the activity of Polo-like kinase 4 (Plk4) and involves the direct interaction of SAS-6 with the centriolar protein SCL/TAL1 interrupting locus (STIL) in vertebrates, or the STIL-equivalent proteins in *Caenorhabditis elegans* (SAS-5) and *Drosophila* (Anastral spindle two; Ana2). Following SAS-6 localization, a scaffold-like structure, referred to as the “cartwheel” in most species (reviewed in Hirono, 2014; Vakonakis, 2020), forms at the onset of organelle assembly. The cartwheel then recruits and organizes further centriole components, including microtubules, thus resulting in the formation of a pro-centriole, which matures into a full organelle in the subsequent cell cycle (Azimzadeh and Marshall, 2010; Firat-Karalar and Stearns, 2014; Gönczy and Hatzopoulos, 2019; Jana et al., 2014). Thus, a single occurrence of pro-centriole formation next to each pre-existing centriole at each cell cycle, coupled to the distribution of two centriolar units to each daughter cell following mitosis, serves to maintain constant organelle number across cell generations. Errors in organelle assembly, which lead to the production of either too many or too few centrioles, as well as structurally aberrant ones, can lead to cell- and organism-level pathologies, including genomic instability, developmental abnormalities, and tumor formation (reviewed in Bettencourt-Dias et al., 2011; Chavali et al., 2014; Gönczy, 2015; Thornton and Woods, 2009; Venghateri et al., 2015).

**Figure 1:**
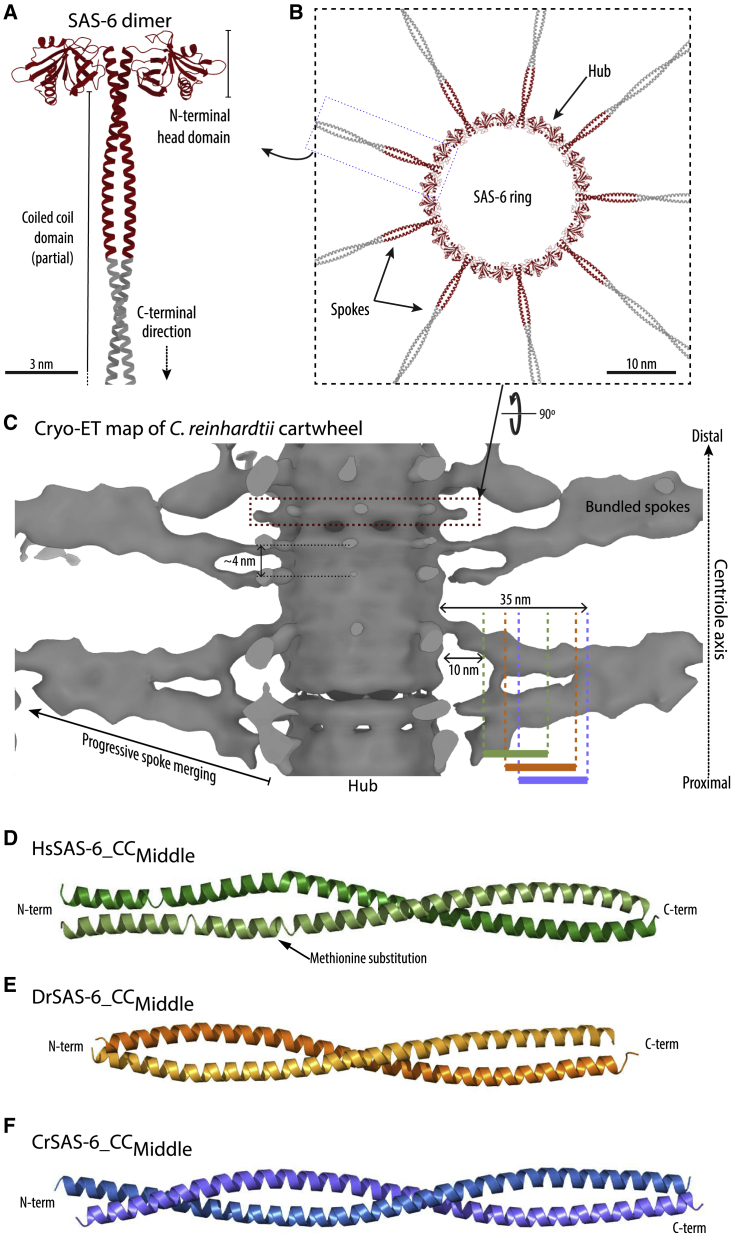
Overview of SAS-6 oligomers in the centriolar cartwheel and of the coiled-coil domains interactions (A) Schematic of SAS-6 dimeric architecture from structural and biophysical studies. Red highlights the region that has been resolved previously with X-ray crystallography, gray a region with unknown structure, modeled here as the continuation of the coiled coil. (B) Nine SAS-6 dimers associate via interactions between their N-terminal head domain to form a SAS-6 ring. (C) Section of the cartwheel from *C. reinhardtii* centrioles resolved by cryo-ET (Klena et al., 2020), showing the central hub and spokes that progressively merge into larger bundles toward the organelle periphery. Red boxed region indicates the position of a single SAS-6 ring. Colored bars mark the regions that have resolved with X-ray crystallography in this study; green bar marks HsSAS-6_Middle_, orange bar DrSAS-6_Middle_, and blue bar CrSAS-6_Middle_. Note that spokes, being initially discrete, have poor density toward the center of the cartwheel. (D–F) Schematic representation of the SAS-6 coiled-coil dimer structures resolved from HsSAS-6_CC_Middle_ (D), DrSAS-6_CC_Middle_ (E), and CrSAS-6_CC_Middle_ (F) crystals and *ab initio* phasing by SeMet incorporation. Lighter and darker colors distinguish the two protein chains in each dimer. The HsSAS-6_CC_Middle_ coiled coil is distorted compared with a canonical CC structure at a region N-terminal to the site of SeMet incorporation (indicated). See also Figures S1 and S2.

Genetic and functional genomics analyses revealed that SAS-6 proteins are critical for centriole assembly in nematodes (Dammermann et al., 2004; Leidel et al., 2005), where they were discovered, as well as in all other eukaryotic species where their function has been tested, including algae (Nakazawa et al., 2007), flies (Dobbelaere et al., 2008), and humans (Strnad et al., 2007). SAS-6 disruption abrogated cartwheel formation and led to the absence of organelle assembly in the vast majority of cases, or to the formation of rare centrioles with diverse radial symmetries. Structural analysis revealed that the SAS-6 architecture is remarkably conserved among species (Figure 1A) (Cottee et al., 2015; Hilbert et al., 2013; Kitagawa et al., 2011; van Breugel et al., 2011; van Breugel et al., 2014), despite relatively low sequence conservation. SAS-6 proteins comprise a globular “head” domain at their N-terminus followed by a long coiled coil that mediates stable protein homo-dimerization (Keller et al., 2014), and a disordered C-terminal extension. Nine copies of the SAS-6 homodimer combine via a self-association interaction mediated by the protein head domain into a ring-like oligomer (Figure 1B). The characteristic shape of the SAS-6 ring led to the proposal that the protein head domains comprise the central “hub” of the centriolar cartwheel, with SAS-6 coiled coils forming the cartwheel “spokes” (Figure 1B) (Cottee et al., 2015; Kitagawa et al., 2011; van Breugel et al., 2011; van Breugel et al., 2014; Yoshiba et al., 2019). In this manner, SAS-6 oligomerization could provide the basis for the 9-fold radial symmetry of the cartwheel and guide the corresponding symmetry of the centriolar cylinder. Consistent with this mechanism, SAS-6 variants disrupting the formation of ring oligomers were unable to support centriole assembly in algae, insects, and human cells (Cottee et al., 2015; Kitagawa et al., 2011; van Breugel et al., 2011; van Breugel et al., 2014). Moreover, protein re-engineering of SAS-6 toward the formation of lower symmetry rings drove assembly of centrioles with 8-fold symmetry in the alga *Chlamydomonas reinhardtii* (Hilbert et al., 2016).

Cartwheels *in situ* (Gibbons and Grimstone, 1960; Klena et al., 2020; Nakazawa et al., 2007) and in purified centrioles (Guichard et al., 2010, 2013, 2017; Klena et al., 2020; Nazarov et al., 2020) comprise stacked layers of SAS-6 ring-containing elements forming a three-dimensional (3D) scaffold (Figure 1C). Recent electron tomographic studies of cartwheels from protozoa, alga, and human centrioles suggest this scaffold is polarized along the proximal-distal axis of centrioles (Klena et al., 2020; Nazarov et al., 2020). However, it remains unclear how multiple SAS-6 rings stack to form the cartwheel or how proximal-distal polarity may emerge. Here, we present structural evidence addressing these key questions. Importantly, our work suggests that ring stacking is mediated by an asymmetric interaction of the SAS-6 coiled-coil domains that together with the rotational offset between consecutive rings could provide the basis for cartwheel polarity.

## Results

### Crystallographic structures of SAS-6 coiled coils reveal new homo-oligomerization interactions

Cryoelectron tomograms (cryo-ET) of *Chlamydomonas reinhardtii* centrioles in the cellular context, as well as of isolated centrioles from diverse species, including humans, showed that SAS-6 rings stack with ∼4 nm average vertical periodicity as measured at the central hub (Figure 1C) (Nazarov et al., 2020; Klena et al., 2020). The close stacking of SAS-6 rings brings to proximity the spokes of the protein, which emanate from the central hub in a radial fashion and correspond to the SAS-6 coiled-coil domain (Figures 1A and 1B). In centriolar tomograms, densities corresponding to the spokes show a slight tilt upward or downward relative to the ring plane and merge progressively into larger entities toward the centriole periphery (Figure 1C). *In vitro* reconstituted cartwheels from purified *C. reinhardtii* SAS-6 also showed an ∼4-nm periodicity at their hub, as well as an indication of spoke merging toward the periphery, suggestive of ring stacking emerging from properties and interactions inherent to SAS-6 (Guichard et al., 2017; Nazarov et al., 2020; Klena et al., 2020). Taken together, these data suggest a model whereby SAS-6 coiled-coil domains from nearby ring oligomers interact in a parallel manner to form spoke bundles, which may participate in the stacking of SAS-6 rings. However, the exact nature of such an interaction has not been resolved.

In order to uncover the structures of SAS-6 spoke bundles at high resolution, we attempted to crystallize the coiled-coil domain of the human, *Danio rerio*, and *C. reinhardtii* variants of this protein (HsSAS-6, DrSAS-6, and CrSAS-6, respectively). Recombinantly produced fragments of the corresponding coiled-coil domains (Figure S1A) were folded, as judged by thermal denaturation experiments monitored by circular dichroism (CD) spectroscopy, and formed dimers in solution size exclusion chromatography–multiangle light scattering (SEC-MALS) assays (Figures S1B–S1D). We succeeded in crystalizing a fragment of the coiled-coil domain from each species, with different boundaries, but spanning approximately the middle one-third of this protein region (henceforth, CrSAS-6_CC_Middle_, DrSAS-6_CC_Middle_, and HsSAS-6_CC_Middle_; see Figure S2A for the protein sequence alignment, Table 1 for a list of recombinant protein constructs, and Figure S1A for SDS-PAGE analysis of these constructs). Given the amino acid boundaries of these protein constructs and assuming canonical two-stranded coiled-coil structures, these fragments should correspond approximately to portions of centriolar spokes starting at 10 to 18 nm radially from the central hub and spanning 15 to 17 nm of the spoke length thereafter (Figure 1C). We resolved the structures of these SAS-6 fragments by X-ray diffraction and *ab initio* phasing using SeMet incorporation (Figures 1D–1F; data quality and refinement statistics are shown in Tables 2 and 3). As the HsSAS-6_CC_Middle_ and DrSAS-6_CC_Middle_ fragments did not originally include methionine residues, one or two amino acids, respectively, were mutated to methionine to allow SeMet incorporation. All three SAS-6 structures revealed two-stranded parallel coiled-coil domains as analyzed by SOCKET (Walshaw and Woolfson, 2001), in agreement with the predicted nature of this protein section. However, the HsSAS-6_CC_Middle_ coiled-coil structure was distorted at a region N-terminal to the site of SeMet incorporation, possibly due to the amino acid mutation necessary to allow SeMet introduction (Figure 1D).

**Table 1:** List of protein constructs

Protein	Construct name	Amino acid boundaries and mutations
*C. reinhardtii* SAS-6	CrSAS-6_CC_Middle_	277-390
*C. reinhardtii* SAS-6	CrSAS-6_CC_LE_	277-390 L306A/E326A
*C. reinhardtii* SAS-6	CrSAS-6_CC_LQ_	277-390 L320A/Q330A
*C. reinhardtii* SAS-6	CrSAS-6_CC_LD_	277-390 L339E/D378N
*C. reinhardtii* SAS-6	CrSAS-6_CC_VD_	277-390 V351E/D361N
*C. reinhardtii* SAS-6	CrSAS-6_NL_	1-503
*C. reinhardtii* SAS-6	CrSAS-6_LE_	1-503 L306A/E326A
*C. reinhardtii* SAS-6	CrSAS-6_LQ_	1-503 L320A/Q330A
*C. reinhardtii* SAS-6	CrSAS-6_LD_	1-503 L339E/D378N
*C. reinhardtii* SAS-6	CrSAS-6_VD_	1-503 V351E/D361N
*H. sapiens* SAS-6	HsSAS-6_CC_Middle_	211-312
*H. sapiens* SAS-6	HsSAS-6_CC_Middle_ L254M	211-312 L254M
*D. rerio* SAS-6	DrSAS-6_CC_Middle_	243-358
*D. rerio* SAS-6	DrSAS-6_CC_Middle_ L286M/L321M	243-358 L286M/L321M

**Table 2:** Crystallographic data collection and phasing statistics

Protein	*CrSAS-6_CC*_*Middle*_*(SeMet)*	*CrSAS-6_CC*_*Middle*_*(Native)*	*HsSAS-6_CC*_*Middle*_*L254M (SeMet)*	*DrSAS-6_CC*_*Middle*_*L286M/L321M (SeMet)*
PDB code	6YRL	6YRN	6YS4	6Z26
Space group	C 1 2 1	P 1 2_1_ 1	P 1 2_1_ 1	P 2_1_ 2_1_ 2_1_
Unit cell: a, b, c (Å)	174.30, 39.74, 88.60	86.51, 39.53, 158.87	40.00, 62.00, 153.75	69.38, 70.31, 98.74
Unit cell: α, β, γ (°)	90.00, 108.91, 90.00	90.00, 101.34, 90.00	90.00, 96.28, 90.00	90.00, 90.00, 90.00
Beamline	DLS/I04	DLS/I04	ESRF/ID29	DLS/I03
Wavelength (Å)	0.97949	0.97950	0.979155	0.97930
Resolution range (Å) High-resolution shell (Å)	83.82–2.34 (2.38–2.34)	84.82–2.43 (2.47–2.43)	57.46–2.11 (2.17–2.11)^a^	57.27–2.31 (2.37–2.31)^b^
R_merge_^c^	0.061 (0.386)	0.154 (0.855)	0.089 (0.917)	0.121 (0.599)
R_meas_^c^	0.067 (0.481)	0.185 (1.065)	0.106 (1.087)	0.127 (0.624)
R_pim_^c^	0.027 (0.280)	0.101 (0.601)	–	–
Completeness^c^ (%)	88.1 (44.2)^d^	99.8 (98.9)	85.1 (27.9)^e^	65.1 (4.2)^f^
Multiplicity^c^	5.5 (2.8)	3.2 (2.9)	6.5 (6.1)	13.0 (12.7)
Mean I/σ(I)^c^	17.5 (2.1)	3.7 (1.1)	7.1 (1.66)	9.9 (3.54)
CC_½_^c^	0.999 (0.895)	0.995 (0.492)	0.988 (0.578)	0.999 (0.887)
*Phasing*				
No. of heavy atoms	10	–	8	10
FOM *initial*	0.31^g^	–	–	0.17^h^
FOM *DM*	0.57^i^	–	0.52^j^	0.30^k^

a Data anisotropically truncated in post-processing by the UCLA diffraction anisotropy server (Strong et al., 2006). Highest resolution diffraction estimated as 2.4 Å, 2.1 Å, and 2.1 Å along a^∗^, b^∗^, and c^∗^ axes, respectively, based on a mean F/σ(F) > 3.0 criterion.

b Data anisotropically truncated in post-processing by the UCLA diffraction anisotropy server (Strong et al., 2006). Highest resolution diffraction estimated as 3.3 Å, 2.6 Å, and 2.3 Å along a^∗^, b^∗^, and c^∗^ axes, respectively, based on a mean F/σ(F) > 3.0 criterion.

c Values in parentheses correspond to highest resolution shell.

d 97% completeness to 2.78–2.76 Å resolution shell.

e 98% completeness to 2.53–2.44 Å resolution shell.

f 98% completeness to 3.27–3.12 Å resolution shell.

g From PHASER (McCoy et al., 2007).

h From REFMAC (Murshudov et al., 1997).

i From RESOLVE (Terwilliger, 2000).

j From SHELXE (Sheldrick, 2010).

k From PARROT (Cowtan, 2010).

**Table 3:** Crystallographic data refinement statistics

Protein	*CrSAS-6_CC*_*Middle*_*(SeMet)*	*CrSAS-6_CC*_*Middle*_*(Native)*	*HsSAS-6_CC*_*Middle*_*L254M (SeMet)*	*DrSAS-6_CC*_*Middle*_*L286M/L321M (SeMet)*
PDB code	6YRL	6YRN	6YS4	6Z26
*Refinement statistics*				
*R*_work_ (reflections)	24.8% (20,678)	28.2% (38,296)	23.9% (35,015)	27.7% (13,434)
*R*_*f*ree_(reflections)	29.7% (1,004)	30.8% (1,973)	28.8% (1,842)	29.7% (708)
*Number of atoms*				
Protein atoms	3382	6527	4901	3302
Ligands	–	7	19	–
Water	74	69	186	25
*Average B factors (Å*^*2*^*)*				
Protein atoms	59.3	49.8	61.9	58.0
Ligands	–	74.4	64.2	–
Water	53.8	28.3	47.0	23.3
*RMSD from ideal values*				
Bonds/angles (Å/°)	0.006/0.68	0.010/1.12	0.010/1.13	0.010/1.19
*MolProbity statistics*				
Ramachandran favored (%)	100.0	99.8	99.6	99.2
Ramachandran disallowed (%)	0.0	0.0	0.0	0.0
Rotamers favored (%)	76.8	91.0	94.5	88.4
Rotamers poor (%)	0.0	0.0	0	0
Clashscore (percentile)	7.11 (98^th^)	3.83 (99^th^)	3.57 (99^th^)	3.71 (99^th^)
MolProbity score (percentile)	1.39 (99^th^)	1.17 (100^th^)	1.15 (100^th^)	1.16 (100^th^)

**Figure 2:**
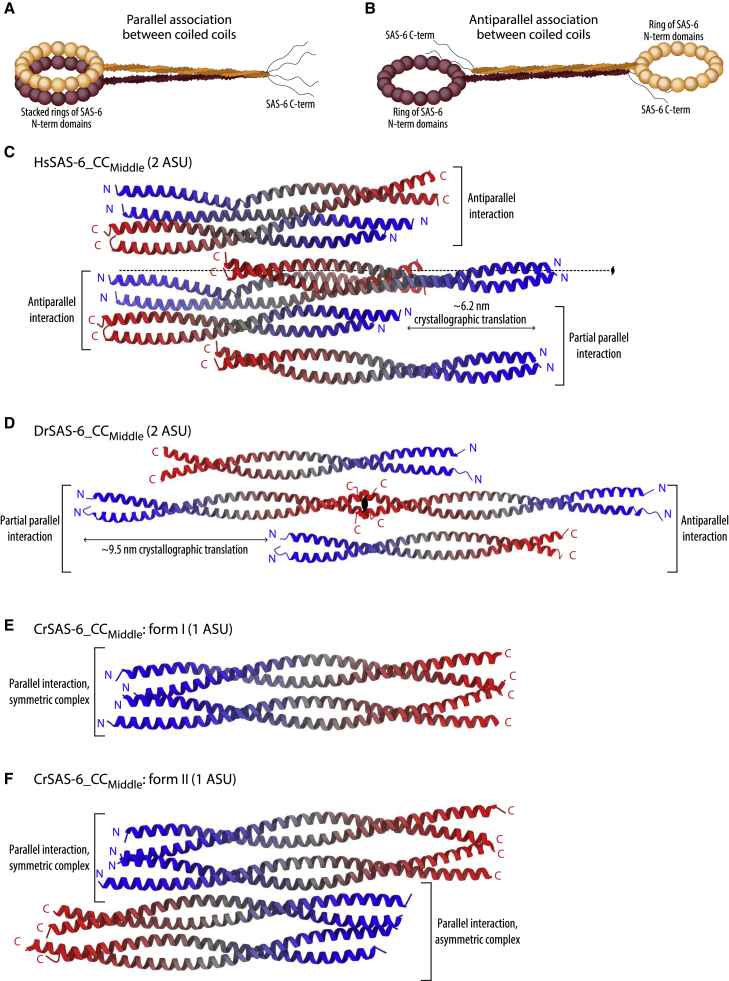
SAS-6 coiled-coil interactions in crystals (A and B) Schematic representation of how SAS-6 rings would position following an (A) parallel or (B) antiparallel association between coiled-coil domains. As shown, parallel association is the only mode compatible with stacking of SAS-6 rings, thereby giving rise to cartwheels. (C, D) Two adjacent asymmetric units from DrSAS-6_CC_Middle_ (C) and HsSAS-6_CC_Middle_ (D) crystals. Chains are colored from N-terminus (blue) to C-terminus (red). (E and F) Similar representation of a single asymmetric unit from CrSAS-6_CC_Middle_ crystal forms I (E) and II (F).

We then analyzed the higher-order interactions between SAS-6 coiled-coil dimers present in the asymmetric units of the crystals as well as across the crystal lattices. As noted earlier, only parallel associations between coiled-coil domains are compatible with the stacking of SAS-6 rings into a continuous cartwheel (Figure 2A), whereas antiparallel coiled-coil interactions would lead to horizontal displacement of SAS-6 rings, a distribution incompatible with a stacked rings configuration (Figure 2B). Both the HsSAS-6_CC_Middle_ and DrSAS-6_CC_Middle_ crystal structures revealed primarily antiparallel associations between their coiled coils (Figures 2C and 2D), with only much smaller parallel associations between coiled-coil copies, which were offset by approximately one-half length or the full length of the crystallographic c-axis, respectively (Table 2). Thus, we judged these parallel interactions as likely crystallographic artifacts, and thereby not relevant for cartwheel assembly.

In contrast, structures of the CrSAS-6_CC_Middle_ fragment, resolved from two different crystal forms, revealed parallel complexes featuring extensive interaction interfaces between coiled-coil domains (Figures 2E and 2F), within each crystal’s asymmetric unit. Analysis of interactions between CrSAS-6_CC_Middle_ fragments across asymmetric units did not show any additional extensive parallel complexes. As parallel CrSAS-6_CC_Middle_ interactions have the potential to be relevant for cartwheel assembly, we focused our analysis on complexes of this protein seen within the asymmetric units of the crystals.

### Symmetric and asymmetric complexes of CrSAS-6_CC_Middle_ domains

**Figure 3:**
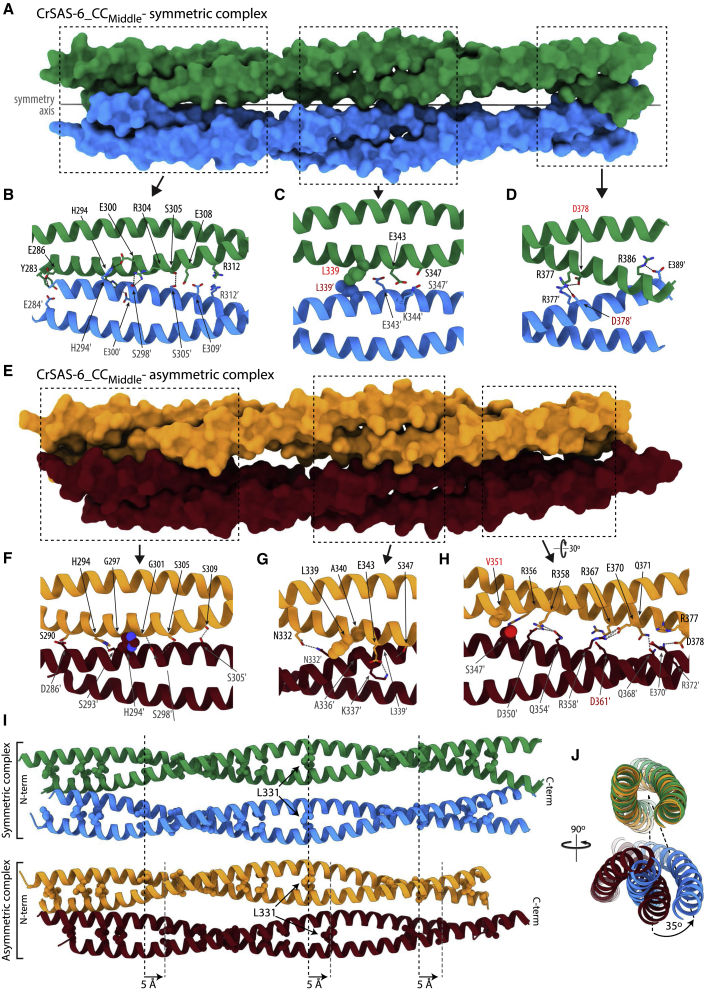
Structures of symmetric and asymmetric complexes of CrSAS-6 coiled-coil domains (A) Surface representation of the CrSAS-6 symmetric complex of two coiled-coil domains, in green and blue, from crystal form I. The internal 2-fold symmetry axis is indicated. (B–D) Detailed views of three key contact areas between the coiled-coil domains. The side chains of amino acids participating in ionic (R312-E309′, E343-K344′, R377-D378′, and R386-E389′, where prime denotes residues of the second coiled coil in the complex) and hydrogen-bonding (Y283-E284′, E300-H294′, R304-S298′, E308-S305′, R312-E309′, S347-S347′, R377-D378′, and R386-E389′) interactions are represented as sticks, and those of a hydrophobic interaction (L339-L339′) as spheres. (E–H) Surface representation of CrSAS-6 asymmetric coiled-coil complex in yellow and red (E) and detailed views of three key contact areas (boxed) in (F–H). Shown in detailed views are amino acids forming key hydrogen-bonding (S290-D286′, H294-S293′, E309-S305′, R314-E308′, N332-N332′, E343-K337′, Q354-S347′, R358-D350′, R358-Q354′, R367-D361′, E370-R358′, Q371-Q368′, D375-R372′, R377-E370′, D378-C373, and D378-R372′), ionic (R314-E308′, E343-K337′, R356-D350′, R358-D350′, R367-D361′, E370-R358′, D375-R372′, R377-E370′, and D378-R372′), and hydrophobic (H294-G297ʹ/G301′, A340-A336′, V351-S347′ interactions. (I) Schematic representation of symmetric (green and blue) and asymmetric (orange and red) CrSAS-6 coiled-coil complexes from crystal form II. The side chains of leucine amino acids in the cores of coiled-coil dimers are shown as spheres for reference. In the asymmetric complex, one coiled-coil dimer is translated by 5 Å (approximately one helical turn) along the longitudinal axis. (J) Overlay of symmetric and asymmetric CrSAS-6 coiled-coil complexes, shown in perpendicular orientation compared with (E) and in a section around L331. One coiled-coil dimer from each complex was superimposed (green and orange); as seen, the second coiled-coil dimers (blue and red) are rotated by 35° relative to one another. See also Figure S2.

In CrSAS-6_CC_Middle_ crystal form I (Figure 2E), resolved to 2.34 Å using SeMet-derived experimental phasing as noted above (Table 2), two copies of the coiled-coil domain in the asymmetric unit interacted over almost their entire ∼16 nm length (Figures 2E, 3A and S2B). The association was driven primarily via ionic and hydrogen-bonding interactions between CrSAS-6 amino acid side chains (Figures 3B–3D), with only a small hydrophobic contribution involving the sidechain of L339 (Figure 3C), resulting in ∼1,400 Å^2^ of buried solvent-accessible surface area. The complex between the two copies of the coiled-coil domains was 2-fold symmetric along its long axis (Figure 3A); thus, most amino acid interactions stabilizing this conformation were observed twice in the crystallographic structure.

CrSAS-6_CC_Middle_ form II, resolved to 2.43 Å using one copy of the CrSAS-6_CC_Middle_ coiled coil from form I as molecular replacement model, featured four copies of the coiled-coil domain per asymmetric unit (Figure 2F). Of these, two copies were arranged in a symmetric-interacting fashion identical to the form I complex (Figures S2C–S2F). In striking contrast, the remaining two CrSAS-6 coiled-coil copies formed a parallel but distinctly asymmetric complex (Figure 3E) driven by hydrogen-bonding and ionic interactions (Figures 3F–3H), which buried ∼1,700 Å^2^ of solvent-accessible surface. The symmetry break was caused by an ∼8-Å shift between the two coiled-coil copies, resulting from a 5-Å translation along the coiled-coil length (Figure 3I) and a 35° relative rotation (Figure 3J). The contact interface of the asymmetric CrSAS-6 coiled-coil complex overlapped to a great extent with that of the symmetric complex, but also involved additional amino acid residues; a comparison of amino acids at the two interfaces is shown in Figure S2B. Hydrophobic contacts were limited to packing of the H294 side chain against G297′ and G301ʹ (Figure 3F, prime denotes amino acids of the second coiled-coil copy in the complex), which form part of a GxxxG motif known for mediating interactions between α-helices (Kleiger et al., 2002), and sidechain interactions between A340-A336ʹ (Figure 3G) and V351 with the aliphatic part of S347ʹ (Figure 3H). Overall, the number and type of amino acid interactions seen in the asymmetric CrSAS-6_CC_Middle_ complex were comparable to those seen in the symmetric complex (Figure S2B).

### Formation of higher-order CrSAS-6 coiled-coil oligomers in solution

**Figure 4:**
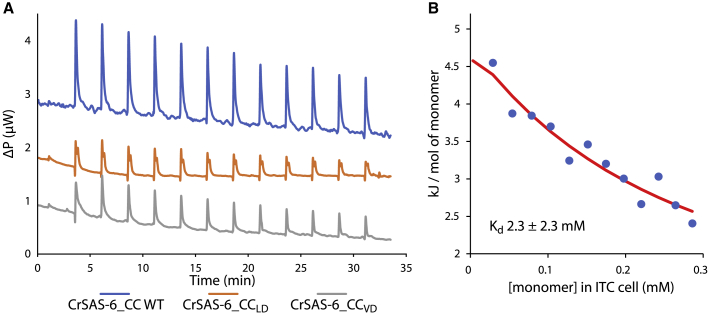
Analysis of CrSAS-6 oligomeric state in solution (A) ITC of CrSAS-6_CC WT, CrSAS-6_CC_LD_, and CrSAS-6_CC_VD_. Concentrated CrSAS-6_CC_Middle_ injected into sample buffer alone produced heat absorptions (ΔP) consistent with the dissociation of a weak protein complex. Substitutions of amino acids affecting either the symmetric (CrSAS-6_CC_LD_) or the asymmetric (CrSAS-6_CC_VD_) interaction mode reduces the observed heats of dilution compared with WT. (B) Analysis of the CrSAS-6_CC_Middle_ dilution heats plot in relation to the monomer concentration suggests that the K_d_ of CrSAS-6_CC_Middle_ coiled-coil dimers is in the low mM range. See also Figure S3.

We sought to evaluate whether higher-order oligomerization between CrSAS-6_CC_Middle_ coiled coils occurs in solution. Our initial SEC-MALS assays, performed as part of recombinant coiled-coil fragments characterization, had failed to detect an association between copies of the CrSAS-6_CC_Middle_ coiled-coil dimer, even under protein concentrations as high as 10 mg/mL (Figure S1D). We surmised that the higher-order oligomerization interactions observed in crystals must be very weak as to not be evident in these SEC-MALS assays. Therefore, we used isothermal titration calorimetry (ITC) instead, which allowed us to work with small volumes of highly concentrated (up to ∼24 mg/mL) CrSAS-6_CC_Middle_ samples; increasing protein concentrations significantly above this point resulted in samples with gel-like qualities unsuitable for quantitative analysis. Concentrated CrSAS-6_CC_Middle_ injected into sample buffer alone produced heats of dilution (ΔH) consistent with the dissociation of a weak protein complex (4.2 ± 0.3 kJ/mol; Figures 4A and S3A), while similar dilution heats were not observed with control buffer (Figure S3B). Analysis of the CrSAS-6_CC_Middle_ dilution heats did not allow accurate determination of the interaction dissociation constant (K_d_), as we were unable to saturate complex formation (Figure 4B); nevertheless, we could conclude that the interaction strength of CrSAS-6_CC_Middle_ coiled-coil dimers is in the mM K_d_-range.

As crystallographic structures resolved both symmetric and asymmetric interaction modes between CrSAS-6_CC_Middle_ coiled-coil dimers, we sought to identify which of these modes is prevalent in solution. To that end, we engineered amino acid substitutions in CrSAS-6_CC_Middle_ aiming to disrupt specific favorable contributions to these interactions. Our freedom to substitute amino acids was restricted, as the two interaction modes occur via largely overlapping protein interfaces (Figures 3I and S2B). We evaluated that an L339E/D378N double mutation of the CrSAS-6 coiled coil (CrSAS-6_CC_LD_) would have the largest impact on the symmetric interaction mode, as these substitutions remove the sole hydrophobic contact seen in the symmetric complex interface (L339-L339′, Figures 3C and S4D), as well as two salt bridges of the same complex (D378-R377′ and the symmetry-related pair, Figures 3D and S4D). In contrast, the same substitutions would eliminate only one salt bridge (D378-R372′, Figure 3H) from the interface seen in the asymmetric CrSAS-6 coiled-coil complex. Similarly, we created a CrSAS-6_CC_Middle_ mutant thought to disrupt primarily the asymmetric coiled-coil interaction seen in crystals. Specifically, we substituted residues V351E/D361N (CrSAS-6_CC_VD_), thereby abrogating a mild hydrophobic (V351-S347′) and an ionic (R367-D361′) contact (Figures 3H and S4E) of the asymmetric interface. None of these amino acids was evaluated as contributing strongly to the symmetric coiled-coil complex. Finally, we engineered two mutants, L306A/E326A (CrSAS-6_CC_LE_) and L320A/Q330A (CrSAS-6_CC_LQ_), that substituted amino acids away from the interaction interface in either the symmetric or the asymmetric CrSAS-6_CC_Middle_ complex, to serve as controls (Figures S4F and S4G).

ITC assays showed that both CrSAS-6_CC_LE_ and CrSAS-6_CC_LQ_ mutants retained an association interaction comparable to wild-type (WT) protein, consistent with their role as controls (Figures S3C and S3D). In contrast, substitutions of amino acids affecting either the symmetric (CrSAS-6_CC_LD_; Figures 4A and S3E) or the asymmetric (CrSAS-6_CC_VD_ Figures 4A and S3F) interaction mode reduced the observed heats of dilution approximately by half compared with the WT protein. As the two mutants had comparable effects on the heats of dilution, we surmised from these assays that both the symmetric and asymmetric interaction modes of CrSAS-6_CC_Middle_ coiled-coil dimers are present in solution, and likely contribute equally to the population of higher-order oligomers.

### Cryoelectron microscopy reconstitution stacking assay supports the role of asymmetric CrSAS-6 complexes in cartwheel formation

We sought to assess the role of SAS-6 coiled-coil complexes in a cell-free cartwheel formation assay, and thereby determine which of the symmetric or asymmetric CrSAS-6 complexes is relevant. A previously developed cryoelectron microscopy (cryo-EM) stacking assay achieved robust reconstitution of cartwheels with a purified CrSAS-6 fragment encompassing both the protein head and full-length coiled-coil domains but lacking the disordered C-terminal tail (CrSAS-6_NL_) (Guichard et al., 2017; Nazarov et al., 2020). In this assay, cartwheels often formed 3D lattices comparable to micro-crystals via contacts between the ends of SAS-6 coiled coils (Figure S5A). To avoid interference from crystallographic packing forces when interpreting the ability of CrSAS-6 mutants to assemble into cartwheels, we focused our analysis on isolated cartwheels that can be observed away from 3D lattices in such a cryo-EM reconstitution assay.

**Figure 5:**
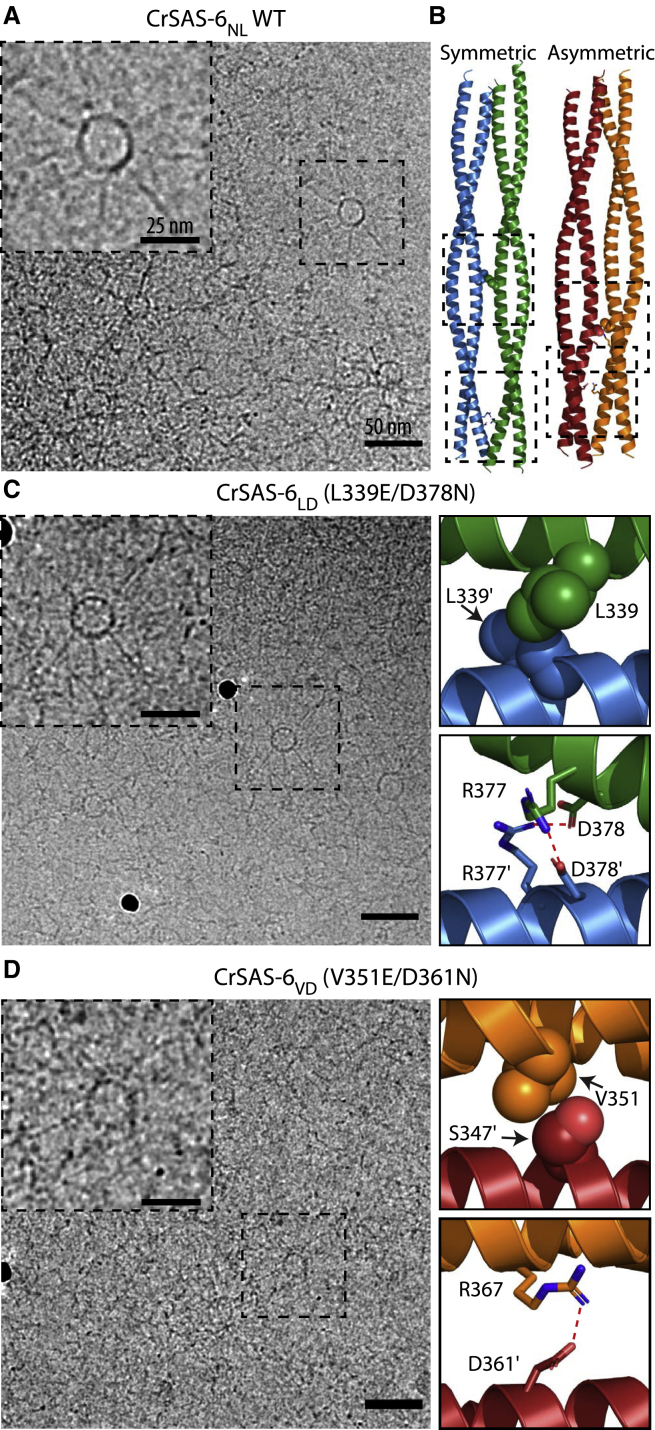
Cartwheel reconstitution using CrSAS-6 WT and mutants (A) Exemplar cryoelectron micrographs of *in vitro* reconstituted cartwheels using WT CrSAS-6_NL_; insets show magnification of field in dashed boxes. (B) Structures of symmetric and asymmetric CrSAS-6 coiled-coil complexes; black dashed boxes denote the areas of amino acid substitutions in (C, D). (C and D) Cryoelectron micrographs of *in vitro* reconstituted cartwheels using CrSAS-6_NL_ mutants designed to destabilize symmetric (C) or asymmetric (D) CrSAS-6 coiled-coil complexes. Structures to the right of micrographs highlight favorable interactions abolished by amino acid substitutions in the symmetric (C) and asymmetric (D) complexes. Red dashed lines in (C, D) indicate hydrogen bonds inferred from the structures. Scale bars 50 nm and 25 nm in the magnification inset. See also Figures S4–S6.

WT CrSAS-6_NL_ readily formed isolated cartwheels with clearly visible hubs and spokes (Figures 5A and S5B). Strong particle contrast (Guichard et al., 2017), as well as the presence of side views (Figure S5B) (Nazarov et al., 2020), indicated that these assemblies corresponded to cartwheel stacks and not simply isolated SAS-6 rings. Particle classification of such assemblies yielded five groups, with most reconstituted cartwheels (80% ± 3% of total) having hub diameter consistent with 9-fold radial symmetry (Figures S5C and S5D), similar to the symmetry of cartwheels in centrioles. Smaller assemblies, with hub diameter consistent with 8-fold radial symmetry, represented a minority of particles (20% ± 3% of total).

We then assessed the formation of cartwheels by CrSAS-6_NL_ fragments bearing mutations on the coiled-coil domain as noted earlier (Figure S4A). CD experiments confirmed that all CrSAS-6_NL_ mutants thus tested remained folded (Figures S4C–S4G). Reconstitution assays using the control mutants L306A/E326A (CrSAS-6_LE_) or L320A/Q330A (CrSAS-6_LQ_) showed clear formation of cartwheels similar to the WT protein (Figures S5E and S5F), with 67% and 84% of particles having diameters compatible with 9-fold symmetry, respectively (Figures S5G and S5H). This result confirmed that these non-interface mutations have little or no effect on cartwheel formation.

We proceeded to test the L339E/D378N (CrSAS-6_LD_) double amino acid substitution, which weakens the coiled-coil association (Figure 4A) and is expected to have a bigger impact in the symmetric than the asymmetric coiled-coil interaction. Thus, CrSAS-6_LD_ should impair cartwheel reconstitution if these assemblies are built primarily through symmetric coiled-coil complexes. In contrast to this prediction, however, reconstitution assays with CrSAS-6_LD_ produced abundant cartwheels (Figures 5B, 5C, S6A–S6C and S6G), similar to those generated using the corresponding WT protein (Figures 5A and S5B–S5D). Furthermore, most cartwheels reconstituted by CrSAS-6_LD_ retained 9-fold radial symmetry (79% ± 12% of particles, Figures S6B and S6C). We surmised that amino acid substitutions that remove favorable interactions of the symmetric CrSAS-6 coiled-coil complex do not affect cartwheel formation *in vitro*.

We performed similar experiments to assess the contribution in cartwheel assembly of amino acids implicated in asymmetric coiled-coil complex formation. We tested the CrSAS-6_NL_ mutant V351E/D361N (CrSAS-6_VD_), which also affects coiled-coil association (Figure 4A), but this time by abolishing interactions of the asymmetric interface. Although CrSAS-6_VD_ did form cartwheels, they were less abundant and less apparent (Figures 5B, 5D, and S6D–S6G) compared with those formed by the WT protein (Figures 5A and S5B–S5D), suggesting reduced stacking propensity. We concluded that amino acid residues contributing favorable interactions in the asymmetric SAS-6 coiled-coil complex likley assist cartwheel assembly *in vitro.*

## Discussion

Centriolar cartwheels have captured the imagination of biologists since their first observation by EM some 60 years ago (Gibbons and Grimstone, 1960). How do these protein assemblies form, and what attributes of centrioles do they contribute to, have been central questions in the field and the focus of research for well over 10 years (reviewed in Hirono, 2014). The present study on self-association interactions of SAS-6, a key cartwheel and centriolar component, advances our understanding over both these questions.

Previous genetic analysis and gene disruption experiments demonstrated that SAS-6 is an essential cartwheel protein (Dobbelaere et al., 2008; Leidel and Gönczy, 2005; Nakazawa et al., 2007; Strnad et al., 2007), and structural studies revealed that it assembles into ring-like oligomers (Cottee et al., 2011; van Breugel et al., 2011; van Breugel et al., 2014; Yoshiba et al., 2019; Nievergelt et al., 2018; Banterle et al., 2021; Kitagawa et al., 2011), which stack into multi-layered cartwheels up to ∼4 μm long in some species (Guichard et al., 2012). What holds SAS-6 rings together into these layered 3D arrangements has been less clear. A previous hypothesis posited that STIL/SAS-5/Ana2, an essential centriolar component that forms higher-order oligomers (Cottee et al., 2015; David et al., 2016; Rogala et al., 2015; Shimanovskaya et al., 2013) and binds to SAS-6 (Dzhindzhev et al., 2014; Hilbert et al., 2013; Ohta et al., 2014; Qiao et al., 2012), could cross-link SAS-6 copies, thereby stabilizing SAS-6 oligomer formation and their 3D arrangement. It is further possible that STIL/SAS-5/Ana2 interactions lead the SAS-6 coiled-coil domains to preferentially interact in parallel, thereby avoiding the antiparallel coiled-coil complexes we observed here in HsSAS-6 and DrSAS-6 crystals (Figures 2C and 2D), and reported previously for *C. elegans* SAS-6 (Qiao et al., 2012). However, purified *C. reinhardtii* SAS-6 protein assembles *in vitro* into structures bearing striking resemblance to centriolar cartwheels (Guichard et al., 2017; Nazarov et al., 2020). Thus, at least some SAS-6 proteins can self-organize into cartwheels comprising coiled-coil moieties in a parallel orientation.

Here, we provide insights toward understanding how SAS-6 rings may stack. In crystallographic structures of the CrSAS-6 coiled coil, we showed that these domains form a unit equivalent to two cartwheel spokes merging together. They do so by associating into a parallel coiled-coil complex (Figures 2E and 2F), the formation of which does not perturb canonical domain structure; for example, the coiled coils do not unwind or adopt a tetrameric coiled-coil arrangement. Rather, complex formation relies on ionic and hydrogen-bonding interactions interspersed along the length of the coiled coils (Figure 3), and is very weak in solution (Figure 4). Interestingly, we note that low ionic strength conditions have long been known as essential for the purification of intact centrioles (Mitchison and Kirschner, 1984), and that *in vitro* reconstitution of cartwheels using CrSAS-6 was only possible in the absence of salts (Guichard et al., 2017; Nazarov et al., 2020); these restrictions may reflect destabilization of the electrostatic bonds mediating SAS-6 coiled-coil interactions by high ionic strength buffers.

How can SAS-6 rings stack through such weak interactions? The answer may be cooperativity of binding, a process also known as avidity, whereby the simultaneous engagement of multiple, individually weak epitopes strengthens the overall interaction affinity (Gwyther et al., 2019; Kitov and Bundle, 2003). Although, in general, full cooperativity is rare due to enthalpic and entropic penalties incurred during simultaneous epitope binding (Andersen et al., 2002), the following considerations enable us to explore how partial cooperativity could contribute to cartwheel formation. CrSAS-6 coiled coils associated with millimolar-level affinity in ITC assays (Figure 4); given that *ΔG=RTln(K*_*d*_*)* (R, natural gal constant; T, temperature), this corresponds to a binding energy ΔG of approximately −15 kJ/mol. Even if only half of this binding energy is retained in a cooperative stacking interaction involving nine coiled coils from two superimposed SAS-6 rings, the resulting sum of binding energies (*ΔG*_*cooperative*_ *= 9 × −7.5 kJ/mol ≅ −68 kJ/mol*) would suffice to produce an apparent interaction affinity between SAS-6 rings (K_d_) of ∼1 pM (*K*_*d*_ *= eˆ(ΔG*_*cooperative*_*/(RT))*. For reference, such an affinity would be comparable to that of the most potent antibody-antigen interactions. Thus, we propose that even modest binding cooperativity between multiple interacting coiled coils could suffice for spontaneous stacking of two SAS-6 rings into a ring-dimer.

Symmetrized cryo-ET reconstructions from native centrioles from several species suggested that spokes of consecutive rings merge toward the cartwheel periphery, with up to six spokes merging into a bundle, as in *C. reinhardtii* (Figure 1C) (Guichard et al., 2012, 2013; Klena et al., 2020; Nazarov et al., 2020; Vakonakis, 2020). More recent analysis of non-symmetrized tomograms of *in vitro* assembled CrSAS-6 cartwheels indicates that such merged spokes do not remain planar on their way toward the periphery (Banterle et al., 2021). Regardless, our findings lead us to propose that spokes from nearby rings are gradually brought together by the association seen in our crystallographic structures (Figures 3A and 3E), being sufficiently close to appear as merged units in tomograms at ∼6 nm from the hub. In this scenario, even though the spokes have the potential to form oligomers larger than tetramers due to their inherent 2-fold symmetry, this does not occur because of the limited lateral distance they can reach given the 4-nm ring periodicity imposed by the central hub. Merged spokes then proceed to the cartwheel periphery, where they attach to the “pinhead” structures that connect spokes to the centriolar microtubules (Hiraki et al., 2007).

**Figure 6:**
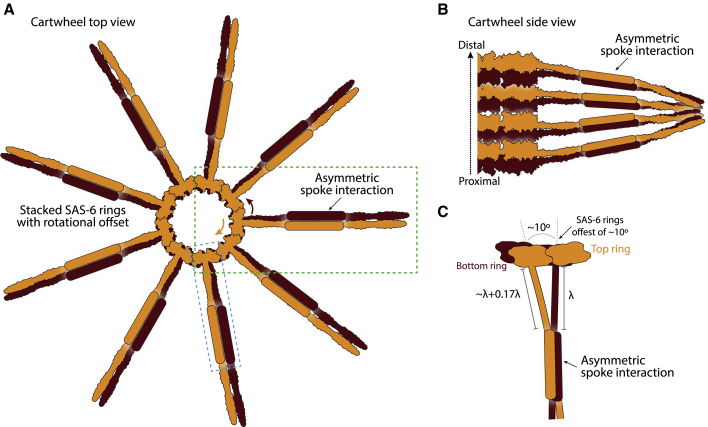
Model of the emergence of centriole polarity rooted in the cartwheel structure (A) Model of SAS-6 ring stacking viewed from the top. Two stacked rings (dark brown at the bottom, orange at the top) are shown schematically, the interacting section of SAS-6 coiled coils are represented as boxes. The rotational offset of the rings as observed in the cryo-ET maps (Klena et al., 2020; Nazarov et al., 2020) is in line with an offset at the coiled-coil merging uncovered here; blue box rotated is shown in (B), green box in (C). (B) Side view of a SAS-6 stack in the cartwheel, showing one-half of CrSAS-6 rings and spokes. The proximal-distal axis of centriole polarity is indicated on the left. Spacings from the centriole center are inferred from crystallographic structures of the CrSAS-6 N-terminal domain defining the hub diameter (Kitagawa et al., 2011), and our coiled-coil structures (this work). (C) Close-up view of the SAS-6 cartwheel model showing how ring offset and the asymmetric coiled coil are in agreement in the current stacking model.

Previous studies considered that an interaction between SAS-6 coiled coils would form the molecular basis of spoke merging (Cottee et al., 2015; Guichard et al., 2017); however, to our knowledge, no study had suggested that merged spokes may be asymmetric. Asymmetrically merged spokes introduce a polar element in cartwheel formation, whereby the relative offset of merged SAS-6 coiled coils provides a directional cue, which might contribute to the proximal-distal directionality of the centriole. Two conditions must be met in order to propagate an asymmetry from the SAS-6 nanometer scale to the organelle micrometer scale. First, centriolar components recruited onto the cartwheel during the organelle assembly process must sustain directionality. Interestingly, peripheral pinhead structures are indeed asymmetric and polarized along the centriole axis (Guichard et al., 2013), such that they could ensure coupling of cartwheel directionality with oriented attachment of microtubules. Second, directional cues imparted by asymmetric spoke merging must be coordinated across all nine spokes to ensure consistent polarity of centriolar microtubules. The random probability of all microtubules spontaneously adopting the correct orientation is just 0.2% (two possible orientations for nine independent sets of microtubules, i.e. one-half^9^). Coordinating directionality across all merged spokes of two consecutive SAS-6 rings could take place via the central hub of each ring, which provides structural connection between adjacent spokes. The hubs of consecutive CrSAS-6 rings could rotate relative to each other, thereby accommodating the 0.5-nm linear offset between coiled coils produced by the asymmetric interaction (Figure 3I). We estimate that a relative hub twist of ∼10° would be sufficient to accommodate this offset (Figures 6A and 6B). Interestingly, this predicted rotation is close to the ∼6.5° twist between successive CrSAS-6 hubs determined from cartwheel tomograms (Nazarov et al., 2020). In the native organelle, perhaps interactions between SAS-6 hubs prime the cartwheel stack with a twist, which is then reinforced by asymmetrically merged spokes; alternatively, this more peripheral asymmetry might drive the central twist. Regardless, coordinated central and peripheral motions would impose the same relative position to all interacting elements from successive SAS-6 ring-containing structures, thereby imparting a single directionality to the cartwheel stack and the emerging centriole (Figures 6A and 6C).

In summary, our work provides structural insight regarding how the spokes of centriolar cartwheels merge to form the basic building block of this large-scale assembly. We demonstrate that the merging interaction is asymmetric, thereby creating a polar structural element in the cartwheel. Consideration of this asymmetric interaction in conjunction with previously visualized structural elements of cartwheels suggests that the central scaffold of centrioles is polar and may define the overall axial polarity of the organelle. Similar to radial symmetry, this cartwheel feature emerges as a consequence of the self-assembly properties of a single centriolar protein, SAS-6.

## STAR★Methods

### Key resources table

**Table:** 

REAGENT or RESOURCE	SOURCE	IDENTIFIER
**Bacterial and virus strains**		

BL21(DE3)	New England Biolabs	Catalog # C2527H
B834(DE3)	Novagen	Catalog # 69041-3

**Chemicals, peptides, and recombinant proteins**		

Gibson Assembly® Master Mix	New England Biolabs	Catalog # E2611L
cOmplete™, Mini, EDTA-free Protease Inhibitor Cocktail	Merck (Roche)	Catalog # 11836170001
Benzonase® Nuclease	Merck (Sigma-Aldrich)	Catalog # E8263-5KU
GST-PreScission protease	Homemade	N/A
Morpheus Complete HT-96	Molecular Dimensions	Catalog # MD1-124
Morpheus® II HT-96	Molecular Dimensions	Catalog # MD1-92
*C. reinhardtii* SAS-6 (277-390)	This paper	N/A
*C. reinhardtii* SAS-6 (277-390; L306A/E326A)	This paper	N/A
*C. reinhardtii* SAS-6 (277-390; L320A/Q330A)	This paper	N/A
*C. reinhardtii* SAS-6 (277-390; L339E/D378N)	This paper	N/A
*C. reinhardtii* SAS-6 (277-390; V351E/D361N)	This paper	N/A
*C. reinhardtii* SAS-6 (1-503)	This paper	N/A
*C. reinhardtii* SAS-6 (1-503; L306A/E326A)	This paper	N/A
*C. reinhardtii* SAS-6 (1-503; L320A/Q330A)	This paper	N/A
*C. reinhardtii* SAS-6 (1-503; L339E/D378N)	This paper	N/A
*C. reinhardtii* SAS-6 (1-503 V351E/D361N)	This paper	N/A
*H. sapiens* SAS-6 (211-312)	This paper	N/A
*H. sapiens* SAS-6 (211-312; L254M)	This paper	N/A
*D. rerio* SAS-6 (234-358)	This paper	N/A
*D. rerio* SAS-6 (234-358; L286M/L321M)	This paper	N/A

**Deposited data**		

CrSAS-6 N-terminal coiled-coil dimer domain	(Kitagawa et al., 2011)	PDB code: 3Q0X
Cryo-ET map of *C. reinhardtii* centrioles	(Klena et al., 2020)	N/A
CrSAS-6_CC_Middle_ (SeMet-derivative)	This paper	PDB code: 6YRL
CrSAS-6_CC_Middle_ (Native)	This paper	PDB code: 6YRN
HsSAS-6_CC_Middle_ L254M (SeMet-derivative)	This paper	PDB code: 6YS4
DrSAS-6_CC_Middle_ L286M/L321M (SeMet-derivative)	This paper	PDB code: 6Z26

**Oligonucleotides**		

Primers used to clone *C. reinhardtii*, *H. sapiens*, and *D. rerio* SAS-6 in pFLOAT2-6xHis are listed in Table S1	This paper	N/A
Primers used for introduction of point mutations in SAS-6 are listed in Table S2	This paper	N/A

**Recombinant DNA**		

Plasmid: pFLOAT2-His	(Rogala et al., 2015)	N/A
pFLOAT2-6xHis-3C-CrSAS-6_CC_Middle_	This paper	N/A
pFLOAT2-6xHis-3C-CrSAS-6_CC_LE_	This paper	N/A
pFLOAT2-6xHis-3C-CrSAS-6_CC_LQ_	This paper	N/A
pFLOAT2-6xHis-3C-CrSAS-6_CC_LD_	This paper	N/A
pFLOAT2-6xHis-3C-CrSAS-6_CC_VD_	This paper	N/A
pFLOAT2-6xHis-3C-CrSAS-6_NL_	This paper	N/A
pFLOAT2-6xHis-3C-CrSAS-6_LE_	This paper	N/A
pFLOAT2-6xHis-3C-CrSAS-6_LQ_	This paper	N/A
pFLOAT2-6xHis-3C-CrSAS-6_LD_	This paper	N/A
pFLOAT2-6xHis-3C-CrSAS-6_VD_	This paper	N/A
pFLOAT2-6xHis-3C-HsSAS-6_CC_Middle_	This paper	N/A
pFLOAT2-6xHis-3C-HsSAS-6_CC_Middle_ L254M	This paper	N/A
pFLOAT2-6xHis-3C-DrSAS-6_CC_Middle_	This paper	N/A
pFLOAT2-6xHis-3C-DrSAS-6_CC_Middle_ L286M/L321M	This paper	N/A

**Software and algorithms**		

autoPROC	(Vonrhein et al., 2011)	https://www.globalphasing.com/autoproc/; RRID:SCR_015748
Aimless	(Evans and Murshudov, 2013)	Run from CCP4 program suite; RRID:SCR_015747
Phenix	(Zwart et al., 2008)	https://www.phenix-online.org/; RRID:SCR_014224
Xia2	(Winter, 2010)	https://xia2.github.io/; RRID:SCR_015746
DIALS	(Winter et al., 2018)	https://dials.github.io/
PHASER	(McCoy et al., 2007)	Run from Phenix suite; RRID:SCR_014219
XDS/XSCALE	(Kabsch, 2010)	http://xds.mpimf-heidelberg.mpg.de/; RRID:SCR_015652
Diffraction Anisotropy Server	(Strong et al., 2006)	https://srv.mbi.ucla.edu/Anisoscale/
SHELX	(Sheldrick, 2010)	http://shelx.uni-ac.gwdg.de/SHELX/; RRID:SCR_014220
Buster	(Bricogne et al., 2017)	https://www.globalphasing.com/buster/; RRID:SCR_015653
CRANK2	(Skubak and Pannu, 2013)	Run from CCP4 program suite
Coot	(Emsley et al., 2010)	http://www2.mrc-lmb.cam.ac.uk/personal/pemsley/coot/; RRID:SCR_014222
Molprobity	(Chen et al., 2010)	Run from Phenix suite; RRID:SCR_014226
ASTRA 6.1	Wyatt Technology	RRID:SCR_016255
Microcal PEAQ-ITC Analysis Software	Malvern	N/A
Epu	ThermoFisher Scientific (FEI)	N/A
Scipion	(Martinez et al., 2020)	http://scipion.i2pc.es/; RRID:SCR_016738
RELION2.0	(Kimanius et al., 2016)	http://www2.mrc-lmb.cam.ac.uk/relion; RRID:SCR_016274
Eman2	(Tang et al., 2007)	https://blake.bcm.edu/emanwiki/EMAN2; RRID:SCR_016867
FiJi	(Schindelin et al., 2012)	http://fiji.sc; RRID:SCR_002285
Illustrator	Adobe	RRID:SCR_010279
PyMOL	(Schrodinger, 2015)	http://www.pymol.org; RRID:SCR_000305
ChimeraX	(Pettersen et al., 2021)	https://www.cgl.ucsf.edu/chimerax/; RRID:SCR_015872

**Other**		

HiTrap Talon	GE Healthcare Life Sciences (Cytiva)	Catalog # 28-9537-67
HiLoad Superdex 75 10/600	GE Healthcare Life Sciences (Cytiva)	Catalog # 28-9893-33
HiLoad Superdex 200 10/600	GE Healthcare Life Sciences (Cytiva)	Catalog # 28-9893-35
Superdex 75 10/300 GL	GE Healthcare Life Sciences (Cytiva)	Catalog # 29-1487-21
Superdex 200 10/300 GL	GE Healthcare Life Sciences (Cytiva)	Catalog # 28-9909-44
Mosquito liquid handling robot	TTP Labtech	N/A
Jasco J-815 spectropolsrimeter	Jasco	N/A
Dawn HELEOS-II	Wyatt Technology	N/A
Optilab-rEX	Wyatt Technology	N/A
Shimadzu HPLC	Wyatt Technology	N/A
Microcal PEAQ-ITC	Malvern	N/A
Slide-A-lyzer MINI, 3.5K MWCO, 0.5mL	ThermoFisher Scientific (Pierce)	Catalog # 88400
300 mesh carbon-coated copper grids	Electron Microscopy Sciences	Catalog # LC325-Cu

### Resource availability

#### Lead contact

Further information and request for resources and reagents should be directed to and will be fulfilled by the lead contact, Ioannis Vakonakis (Ioannis.vakonakis@bioch.ox.ac.uk).

#### Materials availability

Plasmids generated in this study are held by the Department of Biochemistry, University of Oxford, and are available upon request to the Lead contact.

### Experimental model and subject details

Protein expression was performed into *Escherichia coli* strain BL21(DE3) (New England BioLabs) grown at 37°C in lysogeny broth (LB) containing appropriate antibiotics. Expression of SeMet labelled proteins was performed in *E. coli* strain B834(DE3) (Novagen) at 37°C in either LB or SelenoMethionine Medium Base Media (Molecular Dimensions) supplemented with SelenoMethionine Solution (Molecular Dimensions) and appropriate antibiotics.

### Method details

#### Protein production and purification

DNA encoding fragments of *C. reinhardtii*, *Danio rerio*, and human SAS-6 variants (CrSAS-6, DrSAS-6, and HsSAS-6; Uniprot accession numbers A9CQL4, Q7ZVT3 and Q6UVJ0 respectively; Table 1) were cloned into plasmid pFloat2 to include an N-terminal His_6_-tag (Rogala et al., 2015) using Gibson assembly (New England Biolabs). Constructs were transformed into *E. coli* strain BL21(DE3) (New England BioLabs). Transformed clones were grown at 37°C in lysogeny broth (LB) containing appropriate antibiotics until OD_600_ of 0.5-0.7. For CrSAS-6_NL_ WT and mutants the temperature was then switched to 20°C and protein expression induced by the addition of 0.5 mM isopropyl-D-thiogalactoside (IPTG) for 16 h. For SAS-6 coiled coils fragments, 0.3 mM IPTG was added for induction at 37°C for 3.5 h. For expression of SeMet labelled proteins, *E. coli* strain B834(DE3) (Novagen) was used. Cells were grown in LB at 37°C containing appropriate antibiotics to an OD_600_ of 1.0, before harvesting by centrifugation and two washes. Cells were resuspended in sterile H_2_O and used to inoculate SelenoMethionine Medium Base Media (Molecular Dimensions) supplemented with SelenoMethionine Solution (Molecular Dimensions) and appropriate antibiotics. Cells were grown at 37°C to an OD_600_ of 0.6-0.7. The temperature was then switched to 25°C, and expression induced by the addition of 1 mM IPTG for 12 h.

Bacterial cells were harvested by centrifugation. Lysis was achieved by resuspending the bacterial pellet in 50 mM trisaminomethane chloride (Tris-Cl) pH 7.5, 300 mM NaCl buffer supplemented with proteases inhibitors (Complete EDTA-free, Roche) and incubated with Benzonase nuclease (Sigma Aldrich) for 10 min on ice. Sonication was used to lyse resuspended cells, and the resulting lysate was clarified by centrifugation at 48,000 *g* at 4°C. Lysate supernatant was loaded onto a HiTrap Talon metal affinity column (GE Healthcare Life Sciences) pre-equilibrated with lysis buffer supplemented with 20 mM imidazole. Elution was performed with 50 mM Tris-Cl pH 7.5, 300 mM NaCl and 500 mM imidazole buffer followed by His_6_-tag cleavage at 4°C using recombinant human rhinovirus 3C protease. Size exclusion chromatography was performed as a final purification step using Superdex 75 or Superdex 200 columns (GE Healthcare Life Sciences) equilibrated in 50 mM Tris-Cl pH 7.5 and 150 mM NaCl buffer. Protein purity was assessed at each step by SDS-PAGE. Pure protein fractions were extensively dialyzed against 10 mM potassium piperazine-N,N′-bis(2-ethanesulfonic acid (K-PIPES) pH 7.2 buffer, concentrated by centrifugal ultrafiltration with concentrations calculated by UV absorption at 280 nm.

#### Protein crystallisation

Crystals of SAS-6 coiled-coil domain fragments (Table 1) were obtained in SwisSci 96-well plates by the sitting drop vapor diffusion method at 4°C, set up using a Mosquito liquid handling robot (TTP Labtech). Initial crystallisation trials were performed with 200 nL-size drops and 1:1 and 1.3:0.7 protein-to-mother liquor ratios. Initial crystals were obtained using commercial crystallisation screens as outlined below. In order to improve the quality of the crystals we performed crystallisation experiments under the same conditions using seeds obtained from crushing the initial crystals in mother liquor. In microseed crystallisation trials sitting drops consisted of 1:0.5:0.5 and 1:0.3:0.7 protein to seeding stock to mother liquor ratios. Crystals were harvested in nylon loops and flash-cooled in liquid nitrogen.

CrSAS-6_CC_Middle_ fragments produced crystals at 12 mg/mL when mixed with conditions G2 (native protein) or A2 (SeMet-derivatised protein) of the Morpheus I crystallisation screen (Molecular Dimensions). Condition G2 corresponds to 0.1 M of carboxylic acid mixture (sodium formate, ammonium acetate, trisodium citrate, sodium potassium tartrate (racemic), sodium oxamate), 0.1 M imidazole/2-ethanesulfonic acid (MES) buffer pH 6.5, 20% v/v ethylene glycol and 10% w/v polyethylene glycol (PEG) 8000. Condition A2 corresponds to 0.06 M of divalent ions (MgCl_2_, CaCl_2_), 0.1 M imidazole/MES buffer pH 6.5, 20% v/v ethylene glycol and 10% w/v PEG 8000. Crystals appeared after 3 days of incubation and continued to grow over 15 days.

HsSAS-6_CC_Middle_ L254M SeMet-derivatised fragments produced crystals at 6.3 mg/mL when mixed with condition G10 of the Morpheus II crystallisation screen (Molecular Dimensions), corresponding to 100 mM of amino acids II mixture (dl-Threonine, dl-Histidine, dl-5-Hydroxylysine, trans-4-Hydroxy-l-proline), 0.1 M Gly-Gly/2-amino-2-methyl-1,3-propanediol buffer pH 8.5, and 50% v/v of a precipitant mix comprising 25% w/v PEG 4000 and 40% w/v 1,2,6- hexanetriol. Crystals appeared after 2 days of incubation and continued to grow over a week.

DrSAS-6_CC_Middle_ L286M/L321M SeMet-derivatised fragments produced crystals at 9 mg/mL when mixed with condition C6 of the Morpheus II crystallisation screen (Molecular Dimensions), corresponding to 4 mM of alkalis (barium acetate, caesium acetate, rubidium chloride, strontium acetate), 0.1 M BES/Triehylamine buffer pH 7.5, and 50% v/v of a precipitant mix comprising 25% w/v PEG 4000 and 40% w/v 1,2,6- Hexanetriol. Crystals appeared after 2 days of incubation and continued to grow over 10 days.

#### X-Ray data collection, structure solution and refinement

X-Ray diffraction data were collected at beamlines I03 and I04 of the Diamond Light Source (Harwell, United Kingdom), and ID29 of the European Synchrotron Radiation Facility (Grenoble, France) at 100 K. Crystallographic data collection and refinement statistics are shown in Tables 2 and 3. Model quality was assessed by Molprobity (Chen et al., 2010). Molecular representations for figures were created in PyMOL or ChimeraX (Schrodinger, 2015; Pettersen et al., 2021).

Data from CrSAS-6_CC_Middle_ SeMet-derivatised crystals were processed with autoPROC (Vonrhein et al., 2011) and scaled with Aimless (Evans and Murshudov, 2013). The space group was determined to be C 1 2 1 with four protein copies per asymmetric unit of the crystals. *Ab initio* phases were recovered by the SAD method and anomalous signal from incorporated selenium atoms using Phenix.autosol (Zwart et al., 2008). The structure was iteratively refined using Phenix.refine (Zwart et al., 2008) and Coot (Emsley et al., 2010). The crystallographic structure and underpinning data have been deposited in the RCSB Protein Data Bank under accession number 6YRL.

Data from CrSAS-6_CC_Middle_ native crystals were processed with Xia2 (Winter, 2010) and DIALS (Winter et al., 2018), and scaled with Aimless (Evans and Murshudov, 2013). The space group was determined to be P 1 2_1_ 1 with eight protein copies per asymmetric unit of the crystals. Phases were recovered by molecular replacement using PHASER (McCoy et al., 2007) and a copy of the dimeric CrSAS-6_CC_Middle_ coiled coil solved by *ab initio* phasing above. The structure was iteratively refined using Buster (Bricogne et al., 2017) and Coot (Emsley et al., 2010). The crystallographic structure and underpinning data have been deposited in the RCSB Protein Data Bank under accession number 6YRN.

Data from HsSAS-6_CC_Middle_ L254M SeMet-derivatised crystals were processed with XDS (Kabsch, 2010) and scaled using XScale on the UCLA diffraction anisotropy server (Strong et al., 2006). The space group was determined to be P 1 2_1_ 1 with six protein copies per asymmetric unit of the crystals. The structure was solved by *ab initio* phasing using the SAD method and anomalous diffraction signal from selenium atoms using SHELX (Sheldrick, 2010). The structure was iteratively refined using Buster (Bricogne et al., 2017) and Coot (Emsley et al., 2010). The crystallographic structure and underpinning data have been deposited in the RCSB Protein Data Bank under accession number 6YS4.

Data from DrSAS-6_CC_Middle_ L286M/L321M SeMet-derivatised crystals were processed with XDS (Kabsch, 2010) and scaled using XScale on the UCLA diffraction anisotropy server (Strong et al., 2006). The space group was determined to be P 2_1_ 2_1_ 2_1_ with four protein copies per asymmetric unit of the crystals. The structure was solved by *ab initio* phasing using the SAD method and anomalous diffraction signal from selenium atoms using CRANK2 (Skubak and Pannu, 2013). The structure was iteratively refined using Buster (Bricogne et al., 2017) and Coot (Emsley et al., 2010). The crystallographic structure and underpinning data have been deposited in the RCSB Protein Data Bank under accession number 6Z26.

#### Protein biophysical assays

Far-UV CD measurements were conducted using a Jasco (Easton, MD) J-815 spectropolarimeter connected to a Peltier temperature controller. Protein samples were exchanged to a 10 mM K-PIPES pH 7.2, 100mM NaF buffer. Buffer samples without protein were used as base-line measurements. The final ellipticity spectrum was averaged from 4 baseline-corrected measurements to improve signal to noise ratio. Measurements of thermal stability monitored CD signal at 222 nm while temperature was increased by 1°C min^−1^ between 15 and 80°C.

SEC-MALS experiments were performed using analytical Superdex S75 10/300 GL or Superdex 200 10/300 GL columns (GE Healthcare Life Sciences) inline to a Dawn HELEOS-II 8-angle scattering detector and an Optilab-rEX refractive index monitor linked to a Shimadzu HPLC system (Wyatt Technologies, Goleta, CA). Purified samples were injected into a column equilibrated with 10 mM K-PIPES pH 7.2. Data were analysed using the ASTRA 6.1 software package (Wyatt Technology).

ITC assays were performed at 20°C using a MicroCal PEAQ-ITC system (Malvern). Samples of CrSAS-6_CC_Middle_ in 10 mM K-PIPES pH 7.2 buffer and 1.82 mM monomer concentration were loaded in the syringe for stepwise injection into sample buffer alone. Control experiments were performed to assess the contribution of buffer alone in the heat of dilution (buffer-to-buffer injection). The resulting heats were integrated using MicroCal PEAQ-ITC Analysis Software (Malvern) and fit with the dissociation model.

#### *In vitro* cartwheel reconstitution assays

The cryo-EM reconstitution assays were performed as described previously with small modifications (Guichard et al., 2017). Briefly, samples of 20 μL of WT CrSAS-6_NL_ or CrSAS-6_NL_ variants were set for dialysis overnight at 4°C into 10 mM K-PIPES pH 7.2 using 3 kDa molecular weight cut-off slide-A-lyzer mini dialysis units (Thermo Fisher Scientific). From the recovered samples, 5 μL were used for preparing EM grids using a Vitrobot (Thermo Fisher Scientific). The samples were mixed with 15 nm gold nanoparticles and applied on Lacey carbon film grids (300 Mesh, EMS), incubated for 60 s at 5°C, then blotted for 3 s with −15 blot force and vitrified in liquid ethane.

EM was performed on a Tecnai F20 field emission gun electron microscope (Thermo Fisher Scientific) operating at 200 kV and equipped with a Falcon 2 direct electron detector. Images were recorded at × 29,000 magnification in 10 fractions with total dose of 20 e/Å^2^ and automatically aligned by the Epu software (Thermo Fisher Scientific, 0.35 nm final pixel size, −2,5 μm defocus). Areas for imaging were selected randomly in grid squares with similar ice thickness based on the ice quality filter that selects for grid squares with similar range of counts (that are visualised as grey values) over a reference value (I_0_) based on the counts in an image that is acquired at an area where no carbon foil and no ice are present. For each sample, a similar total number of micrographs (∼30 images per replicate) were collected, corresponding to ∼110 μm^2^ in total. This approach was followed to minimise potential differences stemming from the grid preparation process that might lead to partial loss during blotting or from non-uniform sample deposition.

All images were further analysed using Scipion (Martinez et al., 2020). Particles containing top views of CrSAS-6_NL_ rings were picked with RELION2.0 (Kimanius et al., 2016). Particles were classified with RELION2.0 (Kimanius et al., 2016) and Eman2 (Tang et al., 2007). In order to determine particle symmetry, class averages were analysed by performing a line scan with FiJi (Schindelin et al., 2012) and diameters determined by measuring the peak-to-peak distance. Micrographs shown in Figures 5, S5, and S6 were contrast-enhanced by applying bandpass and Gaussian blur filters.

### Quantification and statistical analysis

Average and standard deviation values were determined using AVERAGE and STDEV functions in Microsoft Excel for Figure S6G. Statistical significance was calculated according to a two-tailed Student’s *t* test in Excel. Sample sizes (n) are provided in figure legend. Details for the statistical analysis of X-ray crystallography data are provided above.

## Data Availability

The CrSAS-6_CC_Middle_ form I and form II structure model and associated data have been deposited in the RCSB Protein Data Bank under accession numbers 6YRL and 6YRN, respectively. The HsSAS-6_CC_Middle_ and DrSAS-6_CC_Middle_ structures and data are similarly available under accessions 6YS4 and 6Z26, respectively. This paper does not report original code. Any additional information required to reanalyse the data reported in this paper is available from the lead contact upon request.

## Supplemental information

Document S1. Figures S1–S6 and Tables S1 and S2

Document S2. Article plus supplemental information
