# Supplementary material for: Structures of SAS-6 coiled coil hold implications for the polarity of the centriolar cartwheel
Source: Structure. 2022 May 5;30(5):671–684.e5. doi: 10.1016/j.str.2022.02.005 (PMC9592557; doi:10.1016/j.str.2022.02.005)
Supplement: Document S1. Figures S1–S6 and Tables S1 and S2 [file mmc1.pdf]

Structure, Volume *30*

Supplemental Information

Structures of SAS-6 coiled coil hold implications for the polarity of the centriolar cartwheel

Anastassia L. Kantsadi, Georgios N. Hatzopoulos, Pierre Gönczy, and Ioannis Vakonakis

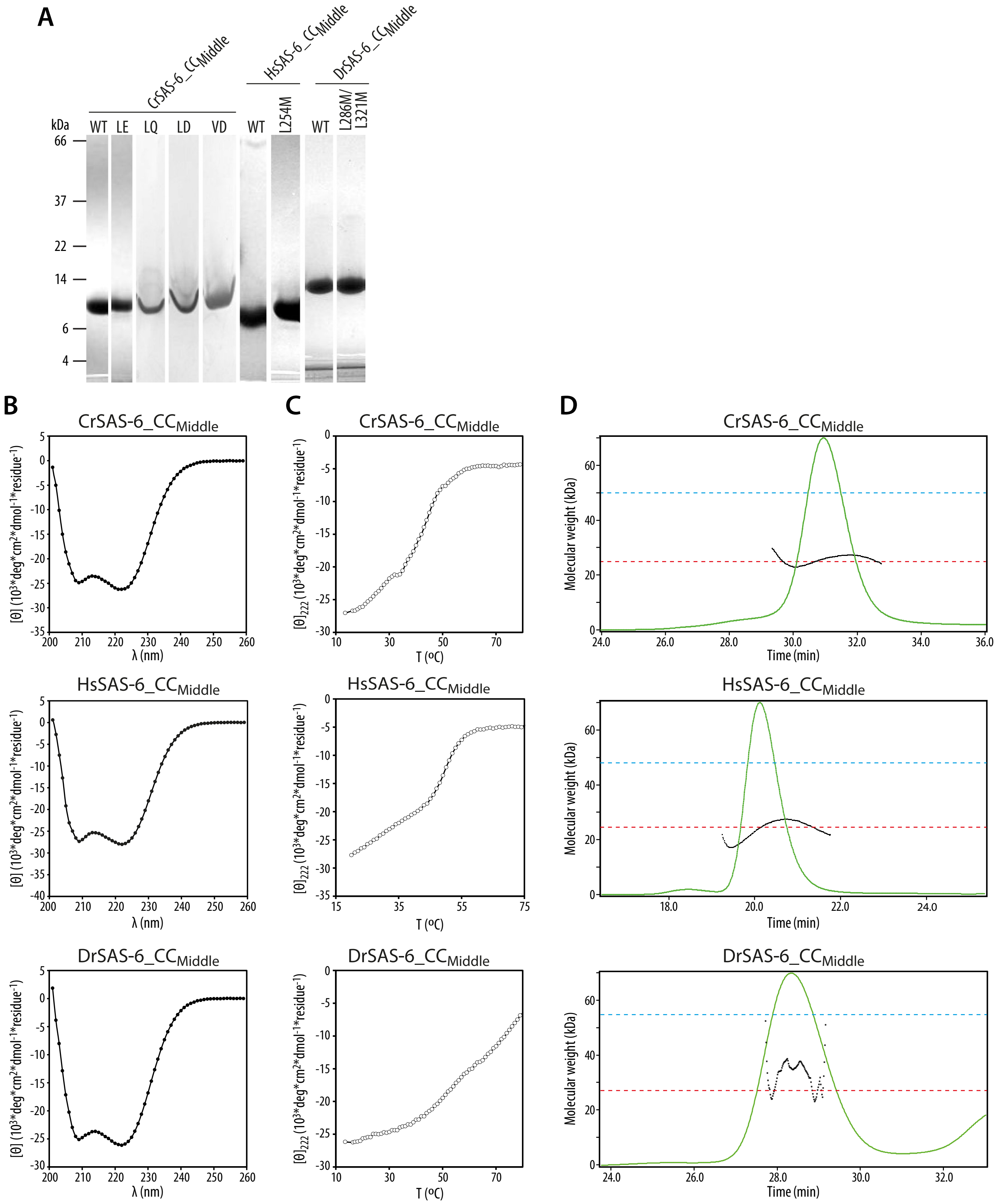

Figure S1. Biochemical and biophysical characterisation of SAS-6 coiled coil domain, Related to Figure 1 and STAR methods.

A) Lanes from SDS-PAGE gels of purified SAS-6_CC proteins. **B-D**) Assessment of SAS-6 coiled-coil folding and oligomerisation. Shown in columns are (B) far-UV CD ellipticity versus wavelength spectra, (C) thermal denaturation profiles monitored by CD at 222 nm as a function of temperature and (D) SEC-MALS elution profiles as a function of time from fragments of the SAS-6 coiled-coil variant indicated at the top. In panels (B) and (C), circles correspond to experimental data points and solid lines to their interpolation. In panels (D), solid green lines correspond to UV absorbance at 280 nm and black lines to estimated molecular weight of the protein species eluting at each time point, plotted on the left axis. Dashed red and blue lines correspond to the calculated molecular weights of dimeric and tetrameric coiled-coil complexes, respectively. Protein sample concentrations for CD were: CrSAS-6_CC$_{Middle}$, 40 µM; HsSAS-6_CC$_{Middle}$, 55 µM; DrSAS-6_CC$_{Middle}$, 35 µM. Sample concentrations for SEC-MALS were: CrSAS-6_CC$_{Middle}$, 10 mg/mL; HsSAS-6_CC$_{Middle}$, 11 mg/mL; DrSAS-6_CC$_{Middle}$, 20 mg/mL.

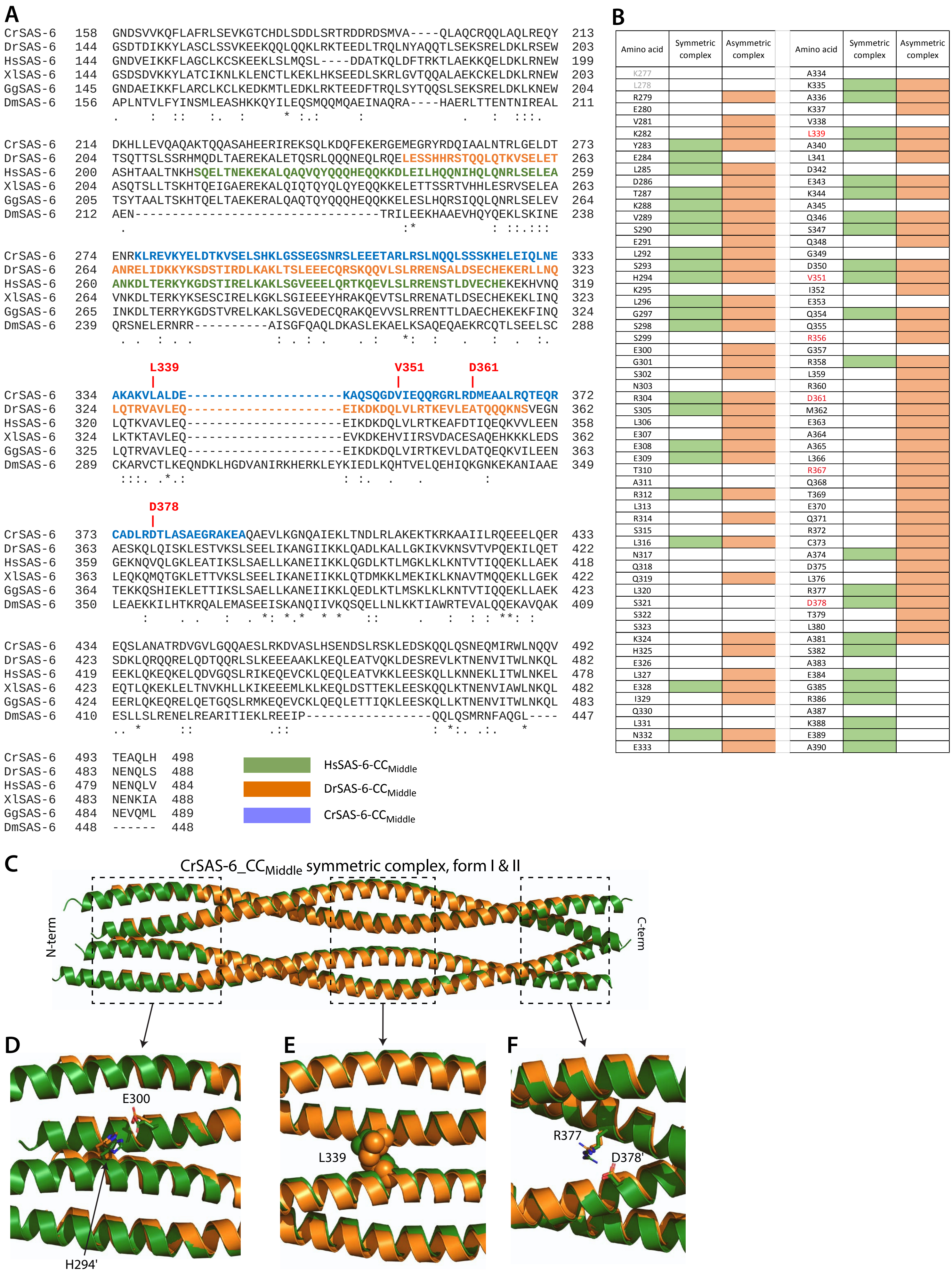

Figure S2. Sequence conservation of the SAS-6 coiled-coil domain and analysis of the interactions in crystals, Related to Figures 1, 2 and 3.

◀ **Figure S2** (cont).

A) Multiple sequence alignment of the coiled-coil region of SAS-6 from *C. reinhardtii*, *D. rerio*, *H. sapiens*, *X. leavis*, *G. gallus*, and *D. melanogaster*. The coiled-coil regions successfully crystallised from *H. sapiens*, *D. melanogaster* and *C. reinhardtii* proteins are highlighted in green, orange, and blue, respectively. Residues important for the coiled-coil interaction in $CrSAS\text{-}6\_CC_{Middle}$ are marked in red. **B**) List of residues of $CrSAS\text{-}6\_CC_{Middle}$ that contribute to the symmetric (green) and/or asymmetric (orange) coiled-coil complex. **C**) Schematic representation of the symmetric $CrSAS\text{-}6\_CC_{Middle}$ complexes as resolved in crystal form I (green) or II (orange). The all-residue Cα root-mean-square-deviation between the two structures is 4.3 Å; this value is inflated by large deviations at the end of the coiled-coil helices. **D-F**) Detailed views of three sets of amino acid interactions stabilising complex formation. Hydrophilic amino acid side chains are represented as sticks (D, F); hydrophobic amino acids as spheres (E). As shown, the structures from both crystal forms feature similar hydrogen bonding (D), hydrophobic (E) and electrostatic (F) interactions.

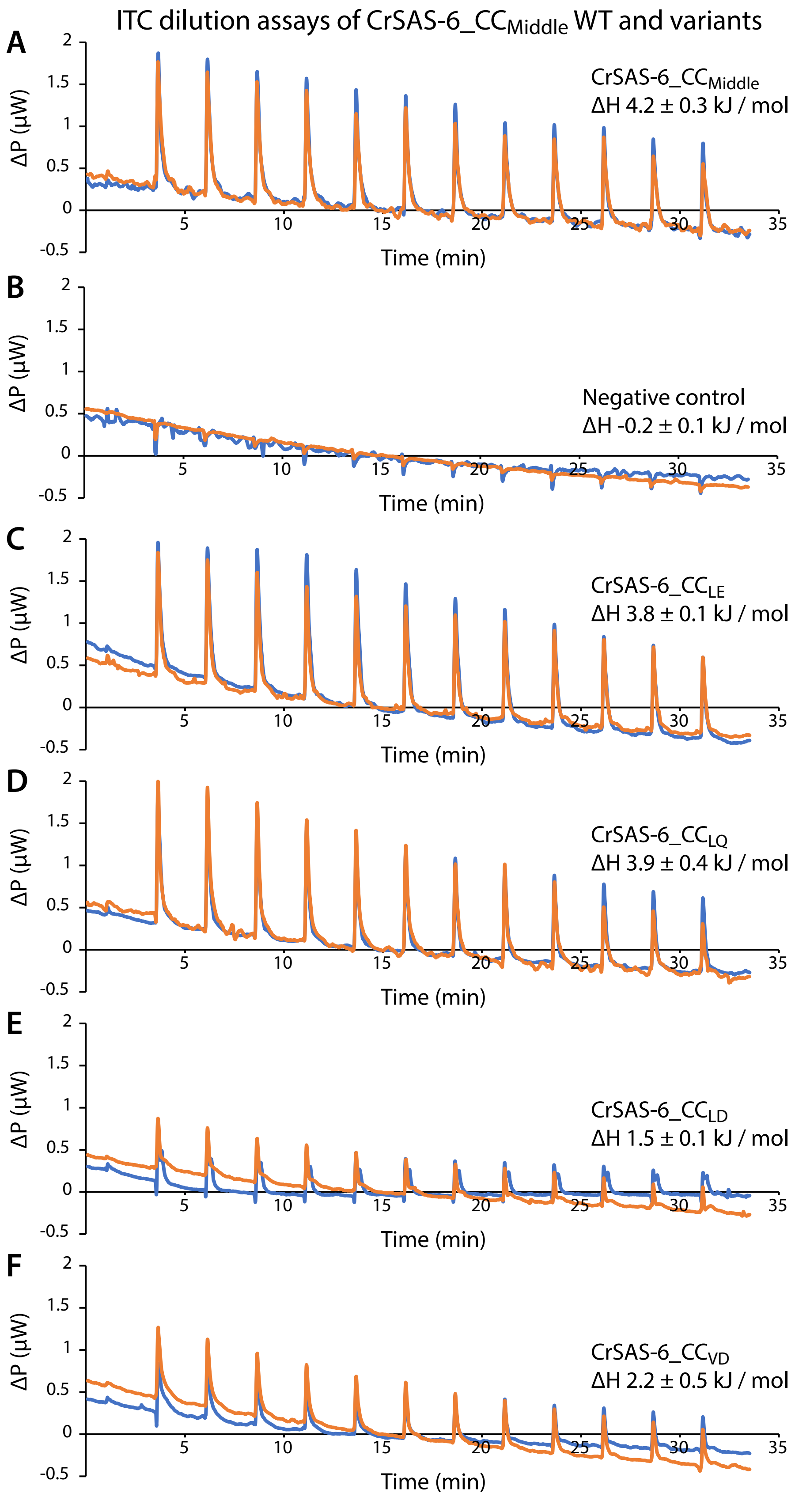

Figure S3. ITC dilution assay of wild-type (WT) and mutant forms of CrSAS-6_CC$_{Middle}$, Related to Figure 4. **A**) Concentrated CrSAS-6_CC$_{Middle}$ WT injected into sample buffer alone produced heats of dilution (ΔP) consistent with the dissociation of a weak protein complex. **B**) Negative control ITC dilution experiment with buffer does not generate heats. **C, D**) ITC dilution experiments with CrSAS-6_CC$_{LQ}$ (C) and CrSAS-6_CC$_{LE}$ (D) showed that they retained an association interaction comparable to WT, consistent with the observation that these residues do not contribute to the coiled-coil interaction. **E, F**) ITC dilution experiments with CrSAS-6_CCMiddle mutants that affect either the symmetric (CrSAS-6_CC$_{LD}$; E) or the asymmetric (CrSAS-6_CC$_{VD}$; F) interaction mode reduced the observed heats of dilution by half or more compared to WT.

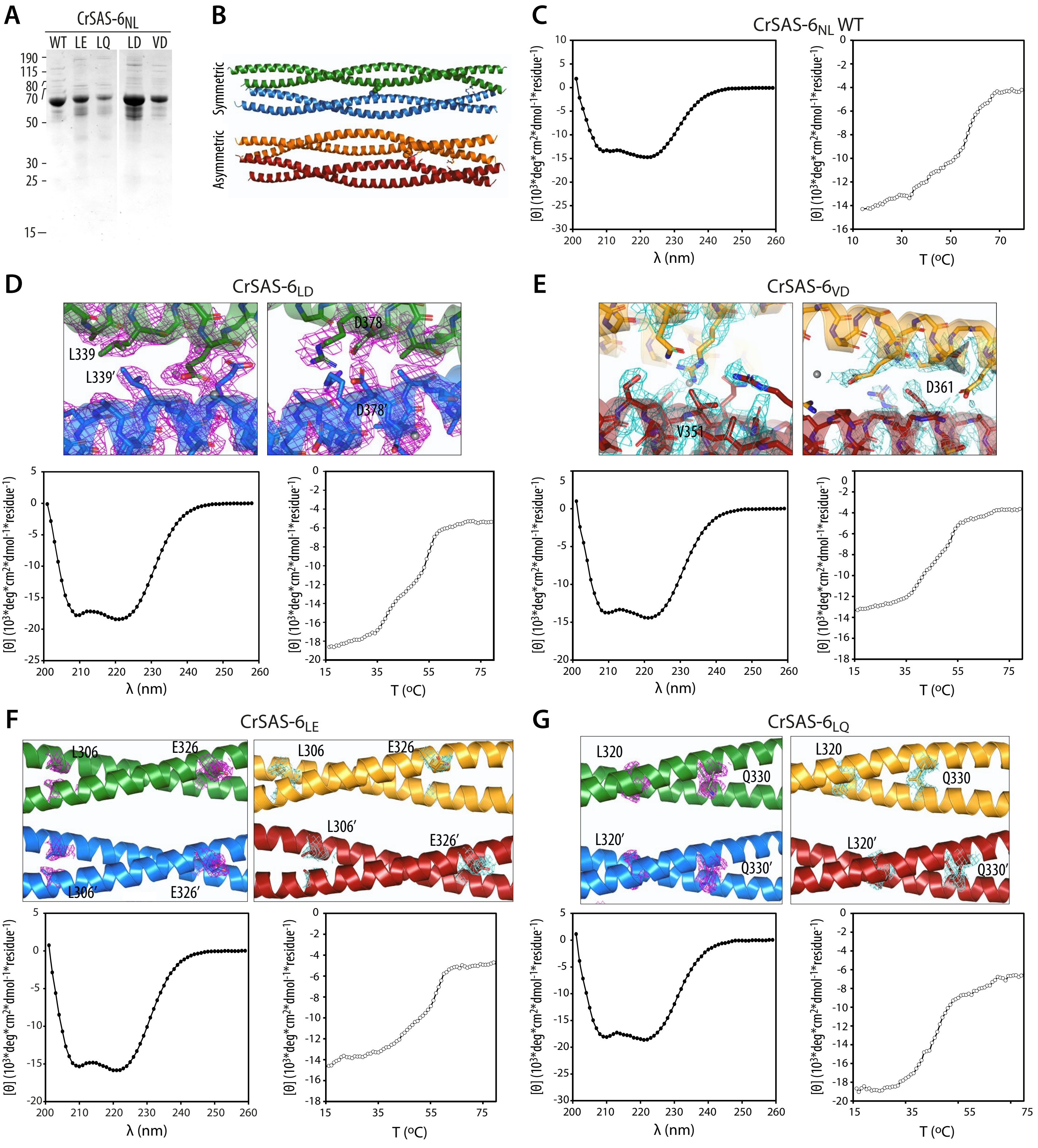

Figure S4. Folding of CrSAS-6$_{NL}$ WT and mutants, Related to Figures 3, 4, and 5.

A) Lanes from SDS-PAGE gels of purified CrSAS-6$_{NL}$ WT and mutant proteins used in the in vitro studies. **B**) Ribbon representation of symmetric and asymmetric CrSAS-6 coiled-coil complexes. **C**) On the left is shown far-UV CD ellipticity versus wavelength spectra, on the bottom right thermal denaturation profiles monitored by CD at 222 nm as a function of temperature for CrSAS-6$_{NL}$ WT (9 µM). Circles correspond to experiment datapoints and solid lines to their interpolation. **D-G**) Biophysical analysis for CrSAS-6$_{NL}$ mutants. In each panel, at the top is shown a ribbon representation of symmetric and asymmetric CrSAS-6 coiled-coil complexes with the density map (2fo-fc at 1σ) of the mutated residues shown in magenta and cyan, respectively. Far-UV CD ellipticity versus wavelength spectra is shown on the bottom left, thermal denaturation profiles monitored by CD at 222 nm as function of temperature on the bottom right for CrSAS-6$_{LD}$ (D, 13 µM), CrSAS-6$_{VD}$ (E, 14 µM), CrSAS-6$_{LE}$ in (F, 9 µM) and CrSAS-6$_{LQ}$ in (G, 13 µM).

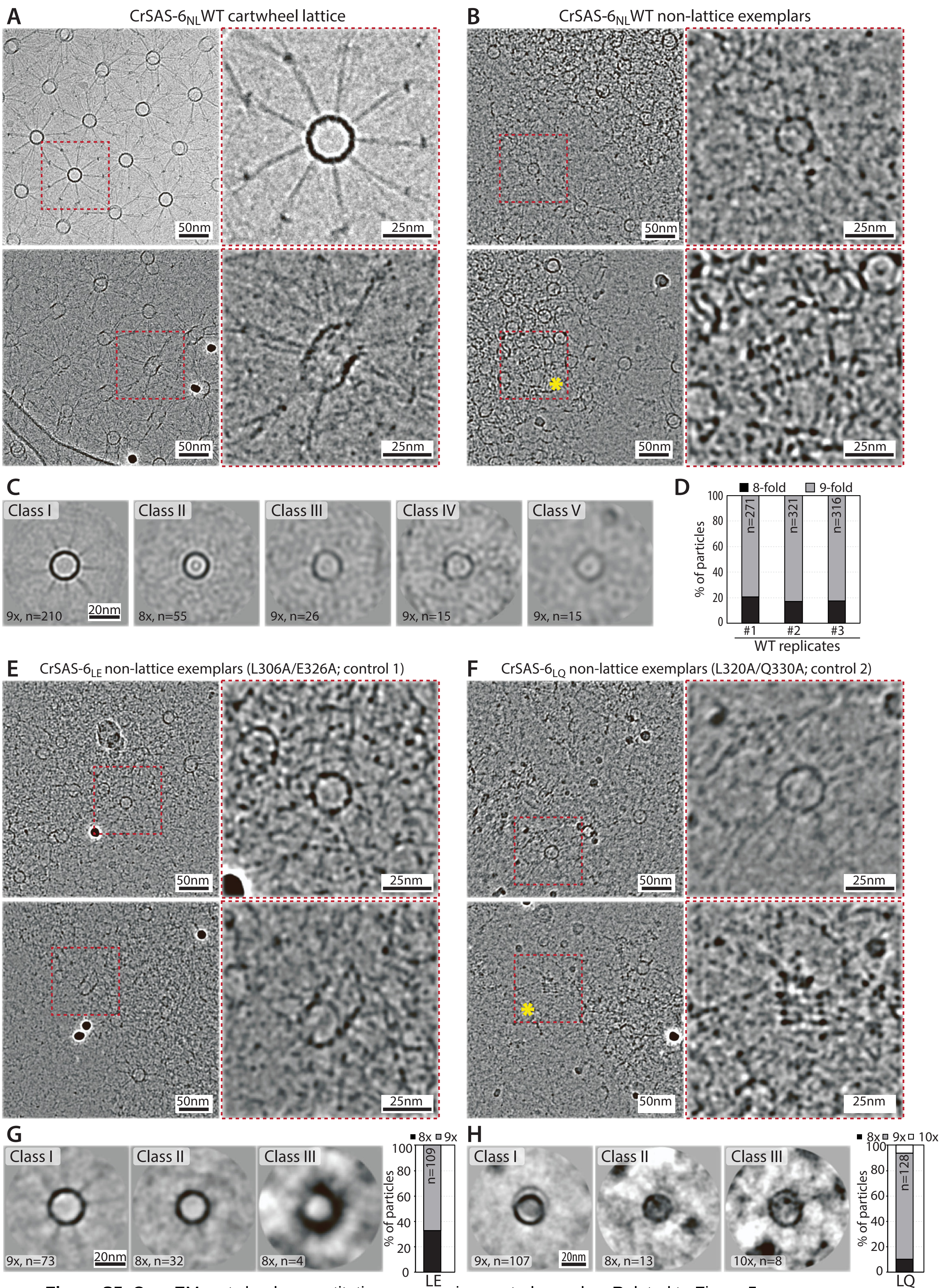

Figure S5. Cryo-EM cartwheel reconstitution assay using control samples. Related to Figure 5.

◀ **Figure S5** (cont).

A, B) Cartwheel reconstitution using wild-type WT CrSAS-6_{NL}. Shown are micrographs from CrSAS-6_{NL} cartwheels in a lattice-like arrangement (A) or as individual entities (B). Red dashed boxes indicate the area magnified on the right. Yellow star indicates a rare side view of an assembled cartwheel. **C**) Class averages of cartwheel particles selected from one reconstitution assay with WT CrSAS-6_{NL}. Particle numbers and radial symmetry of each class, derived from the cartwheel hub diameter, are shown. **D**) Graphical representation of cartwheel symmetry in three independent reconstitution experiments. The number of particles analysed is indicated in the bar graph. **E-H**) Cartwheel reconstitution using control CrSAS-6_{NL} mutants. Similar micrographs and analysis as above for cartwheels reconstituted using CrSAS-6_{LE} (E, G) or CrSAS-6_{LQ} (F, H) mutants. Yellow star indicates a rare side view of an assembled cartwheel.

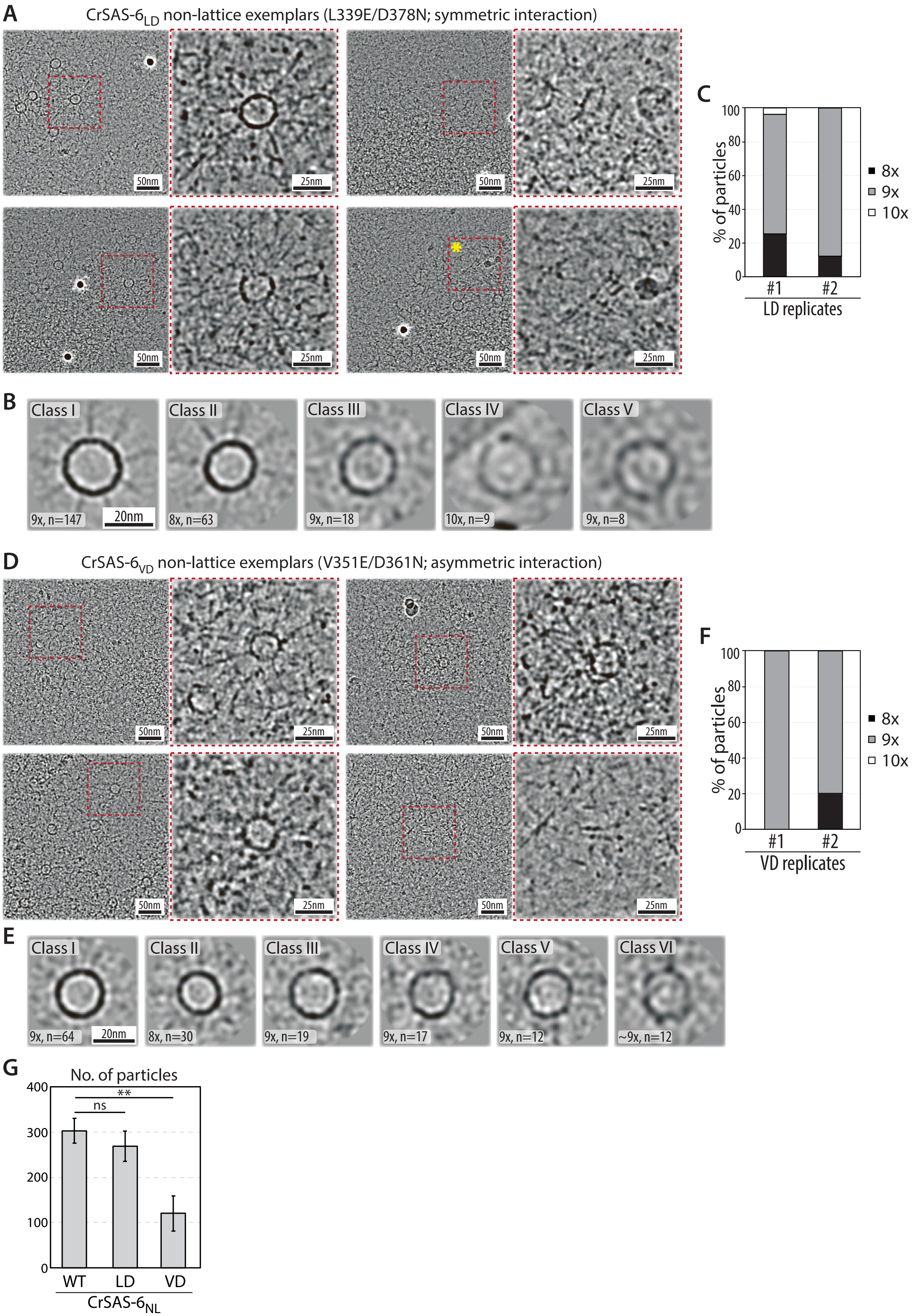

Figure S6. Cartwheel reconstitution using a CrSAS-6NL mutants at the symmetric or asymmetric coiled-coil interface, Related to Figure 5.

Figure S6 (cont).

A) Micrographs from cartwheels reconstituted using the symmetric mutant CrSAS-6_{LD}. Magnified insets in this figure correspond to fields in indicated red dashed boxes. Yellow star indicates a rare side view of the assembled cartwheel. **B**) Class averages of cartwheel particles assembled by CrSAS-6LD. Particle numbers and the radial symmetry of each class, derived from the cartwheel hub diameter, are shown. **C**) Graphical representation of cartwheel radial symmetry in two independent reconstitution experiments for CrSAS-6_{LD}. D) Micrographs from cartwheels reconstituted using CrSAS-6_{VD} and class-averages of these cartwheels (E). **F**) Graphical representation of cartwheel radial symmetry in independent reconstitution experiments for CrSAS-6_{VD}. **G**) Average particle numbers observed for each sample in cartwheel reconstitution assay. Note that this assessment is merely semi-quantitative, as particle numbers can differ in different grid locations (see STAR Methods). Data presented are means ± sd (CrSAS-6_{NL}, n=908 from 3 replicates, CrSAS-6_{LD} n=537 from 2 replicates, CrSAS-6_{VD} n=241 from 2 replicates). Significance according to two-tailed Student's t-test (* $p < 0.05$, ** $p < 0.01$, ***$p < 0.001$).

Table S1. Primers used to clone *C. reinhardtii*, *H. sapiens*, and *D. rerio* SAS-6 in pFLOAT2-6xHis, Related to STAR Methods.

**Oligonucleotide**	**Sequence**
HsSAS-6_CC_{Middle} FRW	TCTGTTCCAGGGGCCCTCTCAGGAACTGACAAATGAAAAG
HsSAS-6_CC_{Middle} REV	TTAGCAGCCGGATCTCTTACTCGTGGCATTCAACATCTA
DrSAS-6_CC_{Middle} FRW	TCTGTTCCAGGGGCCC CTGGAGTCATCTCATCAC
DrSAS-6_CC_{Middle} REV	TTAGCAGCCGGATCTCTTA AGAGTTCTTCTGCTGCTG
CrSAS-6_CC_{Middle} FRW	TCTGTTCCAGGGGCCCCGGGAAGTGAAGTACGAATTA
CrSAS-6_CC_{Middle} REV	TTAGCAGCCGGATCTCTTACGCGCTTCCTTAGCG
CrSAS-6_{NL} FRW	TCTGTTCCAGGGGCCCATGCCGCTTCTTCTCGACG
CrSAS-6_{NL} REV	TTAGCAGCCGGATCTCTCAGGTCGCGCCTGAC

Table S2. Primers used for introduction of point mutations in SAS-6, Related to STAR Methods.

**Oligonucleotide**	**Sequence**
HsSAS-6 L254M FRW	ACAAAACAGAATGTCTGAGTTAG
HsSAS-6 L254M REV	AGCTGGTGGATGTTTTGTTG
DrSAS-6 L286M FRW	CAAAGCCAAAATGACCAGTCTGG
DrSAS-6 L286M REV	AGGTCTCGGATGGTGGAG
DrSAS-6 L321M FRW	GAAGGAGCGTATGCTGAATCAGC
DrSAS-6 L321M REV	TCATGACACTCGCTGTCC
CrSAS-6_CC$_{LE}$ L306A FRW	TAATAGATCGGCGGAGGAGGAAACGG
CrSAS-6_CC$_{LE}$ L306A REV	GAACCTTCGCTAGAGCCT
CrSAS-6_CC$_{LE}$ E326A FRW	CTCAAAGCATGCACTTGAGATAC
CrSAS-6_CC$_{LE}$ E326A REV	GACGACAACTGTTGATTTAAG
CrSAS-6_CC$_{LQ}$ L320A FRW	AAATCAACAGGCGTCGTCCTCAAAGCATGAACTTGAGATAC
CrSAS-6_CC$_{LQ}$ L320A REV	AAGGAGCGTAACCGCGCC
CrSAS-6_CC$_{LQ}$ Q330A FRW	ACTTGAGATAGCACTGAATGAGGCAAAAG
CrSAS-6_CC$_{LQ}$ Q330A REV	TCATGCTTTGAGGACGAC
CrSAS-6_CC$_{LD}$ L339E FRW	AGCAAAAGTCGAGGCCTTGGACG
CrSAS-6_CC$_{LD}$ L339E REV	TTTGCCTCATTCAGTTGTATC
CrSAS-6_CC$_{LD}$ D378N FRW	GGATTTAAGAAACACCCTGGCATC
CrSAS-6_CC$_{LD}$ D378N REV	GCACACCGTTGCTCCGTC
CrSAS-6_CC$_{VD}$ V351E FRW	CCAGGGAGACGAAATAGAACAGC
CrSAS-6_CC$_{VD}$ V351E REV	CTCTGGGCCTTCTCGTC
CrSAS-6_CC$_{VD}$ D361N FRW	GCGGTTAAGAAATATGGAAGCGG
CrSAS-6_CC$_{VD}$ D361N REV	CCGCGTTGCTGTTCTATTAC
CrSAS-6$_{LE}$ L306A FRW	CAACCGCTCGGCGGAGGAGGAGACG
CrSAS-6$_{LE}$ L306A REV	CTGCCCTCCGAGCTACCC
CrSAS-6$_{LE}$ E326A FRW	CTCCAAGCACGCACTAGAGATCCAG
CrSAS-6$_{LE}$ E326A REV	GACGACAGCTGCTGGTTC
CrSAS-6$_{LQ}$ L320A FRW	GAACCAGCAGGCGTCGTCCTCCAAGCAC
CrSAS-6$_{LQ}$ L320A REV	AATGACCGTAGCCGCGCC
CrSAS-6$_{LQ}$ Q330A FRW	GCTAGAGATCGCGCTGAACGAGGCC
CrSAS-6$_{LQ}$ Q330A REV	TCGTGCTTGGAGGACGAC
CrSAS-6$_{LD}$ L339E FRW	GGCCAAGGTGGAGGCGCTGGATG
CrSAS-6$_{LD}$ L339E REV	TTGGCCTCGTTCAGCTGG
CrSAS-6$_{LD}$ D378N FRW	AGATCTGCGCAATACGTTGGCTTCTGC
CrSAS-6$_{LD}$ D378N REV	GCGCACCGCTGCTCCGTC
CrSAS-6$_{VD}$ V351E FRW	GCAGGGCGACGAGATTGAGCAGC
CrSAS-6$_{VD}$ V351E REV	GACTGGGCCTTCTCATCCAG
CrSAS-6$_{VD}$ D361N FRW	ACGCTTGCGTAACATGGAGGCAG
CrSAS-6$_{VD}$ D361N REV	CCACGCTGCTGCTCAATC
